# β3-Adrenergic receptor overexpression in cardiomyocytes preconditions mitochondria to withstand ischemia–reperfusion injury

**DOI:** 10.1007/s00395-024-01072-y

**Published:** 2024-08-12

**Authors:** Miguel Fernández-Tocino, Andrés Pun-Garcia, Mónica Gómez, Agustín Clemente-Moragón, Eduardo Oliver, Rocío Villena-Gutierrez, Sofía Trigo-Anca, Anabel Díaz-Guerra, David Sanz-Rosa, Belén Prados, Lara del Campo, Vicente Andrés, Valentín Fuster, José Luis de la Pompa, Laura Cádiz, Borja Ibañez

**Affiliations:** 1 Clinical Research Department, Centro Nacional de Investigaciones Cardiovasculares Carlos III (CNIC), C/ Melchor Fernandez Almagro 3, 28029 Madrid, Spain; 2 CIBERCV, Madrid, Spain; 3 Centro Nacional de Investigaciones Oncológicas (CNIO), Madrid, Spain; 4 Centro de Investigaciones Biológicas Margarita Salas (CIB), CSIC, Madrid, Spain; 5 Universidad Europea de Madrid (UEM), Madrid, Spain; 6 Universidad Complutense Madrid (UCM), Madrid, Spain; 7 Icahn School of Medicine at Mount Sinai, New York, NY USA; 8 IIS-Fundación Jiménez Díaz University Hospital, Madrid, Spain

**Keywords:** Ischemia–reperfusion injury, Mitochondria, Beta adrenergic receptor, Mitophagy, Preconditioning

## Abstract

β3-Adrenergic receptor (β3AR) agonists have been shown to protect against ischemia–reperfusion injury (IRI). Since β3ARs are present both in cardiomyocytes and in endothelial cells, the cellular compartment responsible for this protection has remained unknown. Using transgenic mice constitutively expressing the human β3AR (hβ3AR) in cardiomyocytes or in the endothelium on a genetic background of null endogenous β3AR expression, we show that only cardiomyocyte expression protects against IRI (45 min ischemia followed by reperfusion over 24 h). Infarct size was also limited after ischemia–reperfusion in mice with cardiomyocyte hβ3AR overexpression on top of endogenous β3AR expression. hβ3AR overexpression in these mice reduced IRI-induced cardiac fibrosis and improved long-term left ventricular systolic function. Cardiomyocyte-specific β3AR overexpression resulted in a baseline remodeling of the mitochondrial network, characterized by upregulated mitochondrial biogenesis and a downregulation of mitochondrial quality control (mitophagy), resulting in elevated numbers of small mitochondria with a depressed capacity for the generation of reactive oxygen species but improved capacity for ATP generation. These processes precondition cardiomyocyte mitochondria to be more resistant to IRI. Upon reperfusion, hearts with hβ3AR overexpression display a restoration in the mitochondrial quality control and a rapid activation of antioxidant responses. Strong protection against IRI was also observed in mice infected with an adeno-associated virus (AAV) encoding hβ3AR under a cardiomyocyte-specific promoter. These results confirm the translational potential of increased cardiomyocyte β3AR expression, achieved either naturally through exercise or artificially through gene therapy approaches, to precondition the cardiomyocyte mitochondrial network to withstand future insults.

**Supplementary Information:**

The online version contains supplementary material available at 10.1007/s00395-024-01072-y.

## Introduction

Acute myocardial infarction (AMI) remains a leading cause of death worldwide [9, 54]. The mainstay therapeutic strategy for AMI is the early restoration or blood flow, known as reperfusion. However, while reperfusion is required for myocardial salvage, it also causes additional specific injury to the myocardium. Thus, the final extent of irreversible damage after AMI is the sum of the injury caused by ischemia and reperfusion, known as ischemia–reperfusion injury (IRI) [6, 7, 38, 39, 42, 47]. Despite major advances in the understanding of IRI, there is still a need to develop new therapeutic strategies to mitigate the impact of AMI [18].

Signaling via members of the G-protein coupled β-adrenergic receptor (βAR) family has been extensively explored as a target for the treatment of several cardiovascular conditions [21, 28, 29, 41, 45, 51]. Although the most highly expressed βAR in cardiomyocytes is the β1 isoform, cardiomyocytes also express the β2 and β3 forms [3, 30]. The β3AR differs from the β1 and β2 isoforms in its downstream signaling, which includes a unique effect on NO production [3]. Treatment with β3AR agonists has been shown to mitigate several forms of myocardial injury, including IRI, heart failure, pressure overload, and pulmonary hypertension with associated right ventricular failure [2, 24–27, 51, 57–59, 63, 65]. The protective effect of β3AR stimulation is amplified by measures to increase cardiac β3AR expression, either through exercise [10, 52] or artificially, either in genetic models or by adeno-associated virus (AAV)-mediated gene therapy [63, 73]. In the heart, βARs are expressed not only by cardiomyocytes, but also in the coronary endothelium [3]. In fact, coronary circulation plays a key role in reperfusion injury [34, 37].

β3AR stimulation in endothelial cells has been shown to improve myocardial neovascularization and left ventricular (LV) function in a model of chronic ischemia [70]. In animal studies showing a benefit of β3AR stimulation in IRI, the β3AR agonist was injected systemically, resulting in stimulation in all cardiac cellular compartments [2, 27]. Since protection against IRI involves crosstalk between the coronary endothelium and cardiomyocytes [36], these in vivo studies do not indicate if the NO-mediated protection upon selective agonist injection during reperfusion is secondary to β3AR stimulation in cardiomyocytes or in endothelial cells. Identifying the cellular compartment involved is a required step toward defining the mechanisms underlying this therapeutic effect and refining strategies for future studies and possible therapeutic approaches.

In the present study, we generated transgenic mice expressing the human β3AR (hβ3AR) only in cardiomyocytes or only in endothelial cells. Our results show that cardiomyocyte but not endothelial-cell β3AR stimulation confers cardioprotection against IRI. Further analysis revealed that cardiomyocyte β3AR overexpression promotes mitochondrial biogenesis and limits fission and mitophagy, resulting in abundant small mitochondria less prone to reactive oxygen species (ROS) production and conferring resistance to apoptosis. These changes protect the heart by preconditioning the mitochondrial network to withstand IRI. IRI mitigation and associated mitochondrial changes were also induced by AAV-mediated cardiomyocyte hβ3AR overexpression in wild type mice, establishing the translational potential of this approach for secondary prevention.

## Methods

### Study design

All experimental and other scientific procedures with animals conformed to EU Directive 2010/63EU and Recommendation 2007/526/EC, enacted in Spanish law under Real Decreto 53/2013. Animal protocols were approved by the local ethics committees and the Animal Protection Area of the Comunidad Autónoma de Madrid. Experiments were conducted with wild-type (Wt) C57BL/6 male mice and male mice of the transgenic lines detailed below on the same C57BL/6J genetic background.

### Transgenic mice expressing the human β3AR

The hβ3AR transgenic mouse line (*ADRB3*^*tg/tg*^) was previously generated by our group [63]. For crosses, we used mouse lines with Cre recombinase expression specifically in endothelial cells (*Tie2*^*Cre/*+^) [44] or cardiomyocytes (*cTnT*^*Cre/*+^) [43]. cβ3Tg mice, with cardiomyocyte-specific overexpression of the hβ3AR in the context of intact expression of the endogenous mouse β3AR (*cTnT *^*Cre/*+^*;ADRB3*^*tg/tg*^) were as previously reported [63]. eβ3Tg mice, with endothelium-specific overexpression of the hβ3AR (*Tie2*^*Cre/*+^*;ADRB3*^*tg/tg*^), were generated by crossing *ADRB3*^*tg/tg*^ mice with the *Tie2*^*Cre/*+^ line. eβ3Tg and cβ3Tg mice were also independently crossed with a knockout mouse line with targeted disruption of the mouse β3AR gene (*Adbr3*): β3KO [72]. These crosses generated mice with the human β3AR solely expressed in endothelial cells (e-restricted-β3, *Tie2*^*Cre/*+^*;ADRB3*^*tg/tg*^*;Adrb3*^*−/*−^), and mice with the β3AR solely expressed in cardiomyocytes (c-restricted-β3, *cTnT*^Cre/+^;*ADRB3*^tg/tg^;*Adrb3*^*−/*−^). Experiments were conducted with male mice at the ages indicated. For controls, eβ3Tg and cβ3Tg mice were compared with their corresponding Wt littermates (*Tie2*^+*/*+^*;ADRB3*^*tg/tg*^ and *cTnT*^+*/*+^*;ADRB3*^*tg/tg*^), and e-restricted-β3 and c-restricted-β3 mice were compared with their β3KO littermates (*Tie2*^+*/*+^*;ADRB3*^*tg/tg*^*;Adrb3*^*−/*−^ *and cTnT*^+*/*+^*;ADRB3*^*tg/tg*^*;Adrb3*^*−/*−^).

### Aorta immunostaining

Paraffin-embedded aortas were cut transversely into 4 mm sections. After antigen retrieval with pH 6.0 citrate buffer, sections were blocked with 10% BSA in phosphate buffered saline (PBS) for 1 h at room temperature and incubated overnight at 4 °C with an anti-GFP primary antibody (Living Colors^®^ Full-Length GFP Polyclonal Antibody, 632,592, Clontech; 1:1000) in PBS. Binding was visualized by incubation with an anti-rabbit peroxidase-conjugated secondary antibody (Horseradish Peroxidase Goat Anti-Rabbit IgG (H&L), ab6721, Abcam; 1:500) in PBS for 60 min at room temperature.

### Blood pressure measurement

Systolic pressure and heart rate measurements were performed in conscious mice using the BP2000 non-invasive automated tail-cuff system (Visitech Systems). All experiments were performed in the morning to avoid variability related to circadian oscillations in the arterial pressure [1, 31]. Animals were trained during 5 days and experimental data were then collected during the next 5 days. At least 10 measurements were registered each day for each mouse. Final measurements were preceded by 10 preliminary measurements to allow the animal to settle. Data are presented as mean from all the measurements of every day. Values equal to 0 were excluded from analysis assuming equipment error or animal movements.

### Myography analysis of ex vivo arterial contractility

Wire myography was performed as previously described [11]. Briefly, thoracic aortas were obtained from 11 to 13-week-old mice and cleaned of fat and connective tissue. Aortas were cut into 2 mm rings, mounted on 2 tungsten wires in a wire myograph system (620M, DMT), and immersed in 37 °C Krebs–Henseleit Solution (115 mM NaCl, 2.5 mM CaCl_2_, 4.6 mM KCl, 1.2 mM KH_2_PO_4_, 1.2 mM MgSO_4_, 25 mM NaHCO_3_, 11.1 mM glucose, and 0.01 mM EDTA) with constant gassing (95% O_2_ and 5% CO_2_). Optimal vessel distension was determined by normalization using the Laplace Equation (below) to calculate the position at which the tension was equivalent to an intraluminal pressure of 100 mmHg (L100).

$${\text{T}} = \frac{{{\text{Pr}}}}{{\text{t}}}$$

where *T* = tension, *P* = pressure, *r* = radius, and *t* = thickness.

Vessels were then set up to the optimal tension (physiological distension, 0.9 of L100), which was maintained for the rest of the experiment. After stabilization for 30 min, arteries were exposed to 120 mM KCl to check functional integrity. Endothelial integrity was checked by examining acetylcholine-dependent vasodilation after precontraction with 1 µM phenylephrine (acetylcholine concentration, 10 µM). The response to β3AR activation was analyzed by recording dose-dependent vasodilation induced with the β3AR agonist mirabegron (0.1–1 µM) after precontraction with 0.1 µM U46619. Data are presented as the percentage of relaxation relative to the initial contraction induced by U4661. To determine the role of NO signaling in mirabegron-induced vasodilation, experiments were also performed in the presence of the specific NOS inhibitor L-NAME (0.1 mM).

### Mouse model of myocardial IR injury

The myocardial IRI protocol and quantification of IS has been previously described [27]. In summary, male 8–12 week-old mice were subjected to occlusion of the left anterior descending (LAD) coronary artery for 45 min followed by reperfusion. Five minutes before the onset of reperfusion, mice were randomized to receive a single bolus injection (50 µl, with an insulin syringe) of mirabegron (1 µg/kg) or saline into the femoral vein. Reperfusion was initiated by release of the suture snare. Animals were recovered with 100% oxygen and analgesized with buprenorphine (0.1 mg/kg) until sacrifice by cervical dislocation.

At 24 h after reperfusion onset, mice were briefly re-anesthetized and re-intubated, and the LAD was re-occluded by ligating the suture in the same position as before. Animals were then killed, and 1 ml of 1% (w/v) Evans Blue dye was infused by retrograde perfusion after aortic cannulation to demarcate the area at risk (AAR). The heart was then excised, the LV was isolated and cut into 7 1 mm-thick transverse slices, and pictures were taken from both sides. To demarcate the infarcted tissue, slices were incubated in 1% (w/v) triphenyltetrazolium chloride (TTC) diluted in distilled H_2_O for 15 min at 37 °C. The slices were then re-photographed from both sides and weighed. For the echocardiography and fibrosis studies mice were sacrificed 7 days after reperfusion onset, and for OROBOROS 2k respirometry, animals were sacrificed 15 min after reperfusion.

### Adult mouse ventricular myocytes isolation

The protocol for adult mpuse ventricular myocytes (AMVM) isolation was as previously described [25]. 10–12 week-old mice hearts were cannulated through the ascending aorta, and mounted on a modified Langendorff perfusion apparatus. The heart was then retrogradely perfused (3 ml/min) for 5 min at RT with prefiltered Ca^2+^-free perfusion buffer (113 mM NaCl, 4.7 mM KCl, 0.6 mM KH_2_PO_4_, 0.6 mM Na_2_HPO_4_, 1.2 mM MgSO_4_-7H_2_O, 12 mM NaHCO_3_, 10 mM KHCO_3_, 0.032 mM Phenol Red, 0.922 mM Na-HEPES, 30 mM taurine, 5.5 mM glucose, and10 mM 2,3-butanedione-monoxime; pH 7.4). Enzyme digestion was performed for 20 min at 37 ºC in digestion buffer [perfusion buffer containing 0.2 mg/ml Liberase (LIBTM-RO, Roche), 2.5% (5.5 mM) trypsin, 5 × 10–3 U/ml DNase, and 12.5 μM CaCl_2_]. At the end of the enzyme digestion, both ventricles were isolated and gently disaggregated in 5 ml digestion buffer. The resulting cell suspension was filtered through a 100 μm sterile mesh (SEFAR-Nitex) and transferred to a tube containing 10 ml stopping buffer-1 [perfusion buffer supplemented with 10% v/v fetal bovine serum (FBS) and 12.5 μM CaCl_2_]. After gravity sedimentation for 25 min, cardiomyocytes were resuspended in stopping buffer-2 (as stopping buffer-1 but with 5% v/v FBS) for another 25 min. Cardiomyocytes were reloaded with Ca^2+^ by 15 min incubations in stopping buffer-2 with five progressively increasing CaCl_2_ concentrations (62 μM, 112 μM, 212 μM, 500 μM, and 1 mM). Resuspension and decanting of cells at each step contributed to the purification of the cardiomyocyte suspension. The homogeneous suspension of rod-shaped cardiomyocytes was then resuspended in M199 supplemented with Earle’s salts and L-glutamine (5 mM), 1% penicillin–streptomycin (P/S), 0.1 × insulin–transferrin–selenium-A, 2 g/l BSA, 25 μM blebbistatin, and 5% FBS. Cells were plated in single drops onto 22 mM 2 glass coverslips precoated with 200 μl mouse laminin (10 mg/ml) in PBS for 1 h.

### Hypoxia/reoxygenation in adult mouse ventricular myocytes

The protocol for hypoxia/reoxygenation in AMCM was performed as previously described [27]. Prior to being subjected to induced hypoxia/reoxygenation, plated isolated adult mouse cardiomyocytes were washed and stabilized for 30 min at 37 ºC with normoxic-buffer (NB) [NaCl (113 mmol/l); KCl (4.7 mmol/l); KH_2_PO_4_ (0.6 mmol/l); Na_2_HPO_4_ (0.6 mmol/l); MgSO_4_·7H_2_O (1.2 mmol/l); NaHCO_3_ (12 mmol/l); KHCO_3_ (10 mmol/l); HEPES-Na Salt (0.922 mmol/l); Glucose (10 mmol/l); CaCl_2_ (1 mmol/l) and pH 7.4]. Hoechst 33,342 (H42, 1 µg/ml) was added for cell recognition, and propidium iodide (PI, 1 µg/ml) was added to evaluate cell viability. Simulated ischemia was induced at 1% O_2_ by placing cells in a H35 hypoxystation chamber (Don Whitley Scientific Limited, UK) in ischemic-buffer (IB), in which glucose and HEPES were replaced with lactate-Na (10 mmol/l) and PIPES (10 mmol/l), at pH 6.8 for 30 min (IB was preequilibrated at 1% O_2_ for 1 h prior to use). After the hypoxia incubation, NB was added on top of IB at a proportion of 1IB:4NB for 1 h to simulate reperfusion. Fluorescent images were acquired with a Nikon Time-lapse microscope after 15, 30, 45 and 60 min of reoxygenation. An average of 350 rod-shaped cells/well observed from 4 wells per condition in 4 independent experiments were analyzed by a blinded using ImageJ 6.0 (NIH, Bethesda, MD, USA). Cell death, indicated by internalization of red fluorescence (Red, PI positive) was expressed as relative cell death compared to the percentage PI positive cell of the total number of cardiomyocytes (Blue, H42 positive) in the wells with cardiomyocytes from β3KO mice at 15 min.

### RNA extraction and cDNA synthesis

Left ventricles from 8 to 12 week-old mice were homogenized in TRIzol Reagent (Invitrogen) for 15 min using a TissueLyser LT tissue homogenizer (Qiagen). Total RNA was extracted with the RNeasy mini kit (74,104, Qiagen) and dissolved in RNAse-free water. Final RNA concentration was measured in a NanoDrop spectrophotometer (Wilmington). cDNA was obtained from RNA with the High-Capacity cDNA Reverse Transcription Kit (Applied Biosystems).

### RT-qPCR

Samples were prepared with 8 ng of cDNA mixed with PowerTrack SYBR Green Master Mix (ThermoFisher) and the following primers: mouse *Adbr3* (Forward: TGATGGCTATGAAGGTGCG; Reverse: AAAATCCCCAGAAGTCCTGC), human *ADBR3* (Forward: TGCCAATTCTTGCCTTCAACC; Reverse: CAGGCCTAAGAAACTCCCCA), mouse *Ucp2* (Forward: AAAGCAGCCTCCAGAACTCC; Reverse: TGTGGCCTTGAAACCAACCA), mouse *Nrf1* (Forward: ACAAGGTGGGGGACAGATAGT; Reverse: ATCTGGACCAGGCCATTAGC), mouse *Sod2* (Forward: CCATTTTCTGGACAAACCTG; Reverse: GACCTTGCTCCTTATTGAAG), mouse *Nox4* (Forward: CGGGATTTGCTACTGCCTCCAT; Reverse: GTGACTCCTCAAATGGGCTTCC), mouse *Cat* (Forward: CTCCATCAGGTTTCTTTCTTG; Reverse: CAACAGGCAAGTTTTTGATG), mouse *Gpx1* (Forward: CGCTCTTTACCTTCCTGCGGAA; Reverse: AGTTCCAGGCAATGTCGTTGCG), mouse *Prkn* (Forward: CCCGGTGACCATGATAGTGTT; Reverse: TGCTGGTGTCAGAATCGACC), mouse *Sqstm1* (Forward: GTGGACCCATCTACAGAGGC; Reverse: GCCTTCATCCGAGAAACCCA), mouse *Becn1* (Forward: AAACCAGGAGAGACCCAGGAG; Reverse: TTCTGTAGACATCATCCTGGCTGG), mouse *Ppargc1a* (Forward: TCCTCTTCAAGATCCTGTTAC; Reverse: CACATACAAGGGAGAATTGC), mouse *Tfam* (Forward: GACCTCGTTCAGCATATAAC; Reverse: ACAAGCTTCAATTTTCCCTG) and mouse *Nrf2* (Forward: GATGACCATGAGTCGCTTGC; Reverse: TATTGAGGGACTGGGCCTGA). Reactions were incubated in a QuantStudio5-384 instrument (ThermoFisher). Gene expression was normalized to mouse *Hprt1* (Forward: AGGGATTTGAATCACGTTTGTGTC; Reverse: TTTGCAGATTCAACTTGCGCT) and mouse *Gapdh* (Forward primer: CATCACTGCCACCCAGAAGACTG; Reverse: ATGCCAGTGAGCTTCCCGTTCAG) and analyzed using the 2^–ΔCt^ and 2^–ΔΔCt^ method.

### Histology and immunohistochemistry

Paraffin-embedded hearts from 8 to 12 week-old mice were cut transversely into 5 µm sections (RM2245 Semi-Automated Rotary Microtome, Leica Biosystems). For fibrosis analysis, sections were stained with 1% Sirius red in picric acid. All sections were scanned with a NanoZoomer-RS scanner (Hammamatsu), and images were exported with NDP.view2. Percentage fibrosis was analyzed with ImageJ. For the determination of mitochondrial area, antigens were retrieved by incubating sections in pH 6.0 citrate buffer, and sections were then blocked by incubation with 10% BSA in PBS for 1 h and incubated overnight at 4 °C with a primary antibody against TOMM20 (1:1000; PA5-52,843, ThermoFisher). Heart sections were then incubated with goat anti-mouse Alexa Fluor Plus 647 secondary antibody (1:1000; A32728, Abcam) in PBS for 1 h at room temperature. Finally, sections were incubated for 15 min with Alexa Fluor 488-conjugated wheatgerm agglutinin (WGA; 1:200, ThermoFisher), followed by 5 min with 4′,6-diamidino-2-phenylindole (DAPI) (1:1000, MERCK), both in PBS at room temperature. Sections were washed 3 times for 5 min in PBS between each step. For apoptosis analysis, In Situ Cell Death Detection Kit, TMR red (12,156,792,910, MERCK) was performed in heart slices according to manufacturer’s protocol. Images were captured with a Zeiss LSM 700 laser scanning microscope and analyzed with ImageJ.

### Echocardiography

Mice were anesthetized with 0.5–2% isoflurane in oxygen delivered via a nose cone, with adjustment to maintain a heart rate of 450 ± 50 bpm. Mice were examined with a 30 MHz transthoracic echocardiography probe and a Vevo 2100 ultrasound system (VisualSonics, Toronto, Canada). A base-apex electrocardiogram (ECG) was continuously monitored through 4 leads. Echocardiography images were analyzed at baseline and 7 days post IRI using the Vevo 2100 Workstation software. For the assessment of LV systolic function, standard 2D parasternal long axis views were acquired at a frame rate > 230 frames/sec. End-systolic and end-diastolic LV volumes (LVESV and LVEDV) and LV ejection fraction (LVEF) were calculated by the area-length method. LV mass was calculated from short axis M-mode views using end-diastolic LV wall thickness.

### Transmission electron microscopy

Transmission electron microscopy (TEM) was performed as previously described [16]. LVs from 8 to 12 week-old mice were fixed in 4% formaldehyde, 1% glutaraldehyde in cacodylate buffer and postfixed in 1% osmium tetroxide. Tissues were washed in PBS, dehydrated through graded alcohols followed by acetone, and then infiltrated with Durcupan ACM Fluka resin and polymerized at 60 °C for 48 h. Sections (60–7 nm) were cut using a Leica ultracut UCT ultramicrotome (Leica, Heerbrugg, Switzerland) and mounted onto 200 mesh grids. Sections were stained with a 2% solution of aqueous uranyl acetate for 10 min, followed by lead citrate staining for 10 min. Treated samples were observed with a JEOL JEM-1010 (100 kV) transmission electron microscope (Tokyo, Japan) at 80 kV through 6000x, 10000x, and 20000 × objectives, and images were acquired with a GATAN Orius 200SC digital camera. Mitochondrial number, area, and perimeter were analyzed using ImageJ (National Institutes of Health, Bethesda, Maryland, USA).

### Protein isolation

LVs from 8 to 12 week-old mice were homogenized using a TissueLyser LT tissue homogenizer (Qiagen) in RIPA buffer (1% Triton 100X, 50 mM tris–HCl pH 7.4, 150 mM NaCl, 0.05% sodium deoxycholate, 0.1% SDS) supplemented with 1 × Complete Protease Inhibitor Cocktail and PhosSTOP phosphatase inhibitors (MERCK) for 15 min. The samples were centrifuged at 12000 rpm at 4 °C for 20 min to remove debris. Final protein concentration was measured using the Pierce BCA Protein Assay Kit (ThermoFisher), with absorbance at 562 nm measured using an xMark Microplate Absorbance Spectrophotometer (Bio-Rad).

### Western blotting

Protein samples (30 μg) were mixed 4:1 (v/v) with 4X Laemmli Sample Buffer (1,610,747, Bio-Rad) and heated at 95 °C for 5 min. Prepared samples were separated by SDS-PAGE and transferred to nitrocellulose membranes (RTA Mini 0.2 μm Nitrocellulose Transfer Kit, Bio-Rad) using a semi-dry transfer system (Trans-Blot Turbo Transfer System, Bio-Rad). Correct protein transfer was confirmed by staining the membranes with the Pierce Reversible Protein Stain Kit for nitrocellulose membranes (ThermoFisher). The membranes were then blocked by incubated for 1 h at room temperature in TBS-t (0.2% Tween-20, NaCl 150 mM, Tris base 20 mM, pH 7.4) containing 5% non-fat milk or 5% BSA (for detection of non-phosphorylated and phosphorylated proteins, respectively). After this, membranes were incubated overnight at 4 °C with primary antibody in TBS-t containing 2.5% BSA Primary antibodies used were to the following targets: vinculin (1:10000; V4505, Sigma), GAPDH (1:10000; ab8245, Abcam), citrate synthase (1:1000; 14,309, Cell Signaling), PGC1-α (1:1000; NBP104676, Novus Biologicals), UCP2 (1:1000; 89,326, Cell Signaling), SOD2 (1:1000, GTX116093, GeneTex), BAX (1:1000; M00183-1, Boster), Bcl-2 (1:1000; E-AB-22004, Elabscience), Parkin (1:1000; 4211, Cell Signaling), PINK1 (1:1000; 6946, Cell Signaling), LC3B (1:1000; 2775, Cell Signaling), sequestosome-1 (p62) (1:1000; 5114, cell signaling), Beclin-1 (1:1000; 3738, Cell Signaling), Fis1 (1:1000; ab229969, Abcam), MFN1 (1:1000; ab126575, Abcam), MFN2 (1:1000; ab56889, Abcam), OPA1 (1:1000; PA1-16,991, ThermoFisher), Yme1L (1:500; PA5-43,806, ThermoFisher), OMA1 (1:1000; ab154949, Abcam), Drp-1 (1:1000; 8570, Cell Signaling), and phospho-Drp-1 (ser616) (1:1000; 3455, Cell Signaling). Washed membranes were then incubated for 1 h with HRP-conjugated goat anti-mouse secondary antibody (1:10000; Immunoglobulins) or goat anti-rabbit secondary antibody (1:5000; HRP-Dako company), as appropriate, in 1:5 of the blocking buffer. Membranes were washed 3 times for 5 min in TBS-t between each step. Signal was developed after incubating membranes with Immobilon Western Chemiluminescent HRP Substrate (MERCK), and images were acquired with an ImageQuant LAS 4000 mini Biomolecular Imager (GE-HealthCare). Protein expression was analyzed with ImageJ and normalized to that of vinculin, GAPDH or total protein quantification. Outliers determined by Grubbs’ test and artifacts were excluded from data. Uncut western membranes are shown in Suppl. Fig. 10 and Suppl. Fig. 11.

### Isolation of mitochondria

LVs were isolated from 8 to 12 week-old mice at baseline (i.e. homeostatic conditions) and after 45 min of ischemia followed by 15 min of reperfusion. Each LV was placed in a glass-tube homogenizer in 2 ml ice-cold isolation buffer (IB; 275 mM sucrose, 20 mM Tris base, 1 mM EGTA, pH 7.2) containing 100 μl/ml trypsin (X0930, Dutscher). Trypsin was inactivated by the addition of 8 ml IB supplemented with 0.025% fatty-acid-free BSA (A6003, Sigma-Aldrich). Samples were centrifuged at 1000xg at 4 °C for 10 min and the supernatants were collected and centrifuged at 3220xg at 4 °C for 10 min. Mitochondrial pellets were carefully resuspended in 100 μl IB + BSA, and protein concentration was measured using the Pierce BCA Protein Assay Kit (ThermoFisher), with absorbance at 562 nm measured using an xMark Microplate Absorbance Spectrophotometer (Bio-Rad).

### High resolution respirometry

Mitochondrial respiration was measured in real time by high-resolution respirometry using an Oxygraph-2K instrument (OROBOROS Instruments, Innsbruck, Austria). The Oxygraph-2K apparatus consists of a 2-chamber respirometer equipped with a Peltier thermostat and electromagnetic stirrers. The oxygen electrodes were calibrated daily at air saturation, with both chambers fully open (set at 2 ml), the temperature set at 37 °C, and stirring at 750 rpm until a stable signal was obtained. Measurements were made in suspensions of freshly isolated mitochondria at a final concentration of 0.3 mg/ml in MiRO5 medium (0.5 mM EGTA, 3 mM MgCl_2_-6H_2_O, 20 mM taurine, 10 mM KH_2_PO_4_, 20 mM HEPES, 200 mM sucrose, and 1 g/l BSA, adjusted to pH 7.1 with KOH), or 5 × 10^5^ isolated cardiomyocytes in 100 μl of MiRO5 medium. The protocol for measuring mitochondrial chain respiratory states was as follows. After closing the chamber, an initial state 2, or routine state, was measured after addition of malate and pyruvate (5 mM each), which are substrates of complex I (CI); this state corresponds to basal respiration in the absence of ADP. Active respiration (state 3) was then initiated by adding 2.5 mM ADP to promote a rapid response of the electron transport chain (ETC) coupled to oxidative phosphorylation (OXPHOS_CI_), which translates into a rapid increase in oxygen consumption until all the ADP is phosphorylated to ATP. ATP synthase was then inhibited by the addition of oligomycin (10 nM), initiating the mitochondrial respiratory state 4 or leak state, which is a non-phosphorylating resting state. After this, titrations of FCCP (1 μM) were added to measure maximum electron transfer capacity (ETC_CI_). Finally, complex III activity was inhibited by the addition of 2.5 μM antimycin A. The respiratory control ratio (RCR, calculated here as OXPHOS/Leak) provides a measure of the degree of coupling between oxidation and phosphorylation, or, in other words, the efficiency of mitochondrial ATP production. Data were obtained at 0.2 s intervals using a computer-driven data acquisition system (Datlab, Innsbruck, Austria).

### ATP quantification

ATP levels were measured in heart samples using an EnzyLight^™^ ATP Assay Kit (EATP-100, BioAssay Systems), performed according to the manufacturer’s instructions. ATP concentrations (μM) were then normalized by μg of heart tissue protein.

### AAV production and in vivo delivery

Adeno-associated virus particles were produced as previously described [63]. Male mice aged 6–8 weeks were anesthetized and maintained on 1–2% isoflurane in oxygen. A dose of 3 × 10^11^ viral genomes (vg)/mouse in 50 μl saline was injected using a 31G insulin syringe though one of the femoral veins. AAV-9 was used for in vivo delivery. At 2 weeks after infection, mice were subjected to 45 min of LAD coronary artery occlusion followed by reperfusion as described above. AAR and IS were determined 24 h after reperfusion. Correct viral transfection was verified by RT-qPCR as described above.

### In vivo autophagic flux quantification

Male 6 week-old mice received a daily oral dose of 1.15–2.02 mg chloroquine (0.288 mg/ml dissolved in tap water; PHR1258-1G, Merck) together with glucose (15 g/l; 108,342, Millipore) to encourage consumption; control mice were given glucose alone [48]. After 4 weeks of chloroquine treatment, mice were subjected to the standard 45 min ischemia—reperfusion protocol described above. AAR and IS were determined 24 h after reperfusion. To evaluate the autophagic flux, 8–12 week-old mice were randomized to receive a single intraperitoneal dose of leupeptin (L2884, Merck) or saline, or chloroquine (PHR1258-1G, Merck) or saline, and LV were collected 45 min or 4 h thereafter, respectively.

### Statistics

Experimental data are represented as mean ± standard deviation (SD) and were analyzed using GraphPad Prism (Graph pad, Inc.). The Mann–Whitney test was performed for comparison of 2 groups, and the Kruskal–Wallis test was performed for comparisons between 3 and more groups. Comparisons between 2 groups in response to increasing drug doses or exposure times were made by two-way ANOVA with Sidak’s multiple comparisons test. A power analysis was used to calculate sample sizes providing statistical significance at a *p*-value ≤ 0.05.

## Results

### Generation of transgenic mice expressing human β3AR in endothelial cells and with disruption of the mouse β3AR gene in all cellular compartments

We previously reported the generation and characterization of mice with cardiomyocyte-specific hβ3AR expression on a background of intact endogenous β3AR expression (cβ3Tg) or disrupted endogenous expression (c-restricted-β3) [63]. Here, we used the endothelial-cell–specific Tie2-Cre line to generate mice with similarly targeted expression of hβ3AR in endothelial cells (eβ3Tg and e-restricted-β3). Tie2-Cre is active in the vascular endothelium from day 7.5 of embryonic development [44]. Confocal imaging of E9.5 eβ3Tg embryos (*Tie2*^*Cre/*+^*;ADRB3*^*tg/tg*^) showed expression of the eGFP reporter gene in the vasculature (Suppl. Fig. 1A), and aortic immunostaining confirmed exclusive expression of hβ3AR in endothelial cells (Suppl. Fig. 1B). To exclude any influence from endogenous β3AR, we crossed eβ3Tg mice with mice with targeted disruption of the mouse β3AR gene (*Adrb3*) to generate e-restricted-β3 mice (*Tie2*^*Cre/*+^*;ADRB3*^*tg/tg*^*;Adrb3 *^*−/−*^). Automated tail-cuff monitoring in conscious e-restricted-β3 mice and β3KO controls revealed that endothelial hβ3AR expression had no effect on basal systolic arterial pressure and pulse (Suppl. Fig. 1C). Ex vivo analysis revealed a vasodilatory response of e-restricted-β3 aortic rings to increasing doses of the β3AR agonist mirabegron, whereas β3KO rings were unresponsive (Suppl. Fig. 1D). Mirabegron-induced relaxation was abolished by addition of the nitric oxide synthase (NOS) inhibitor L-NAME, indicating functional coupling of hβ3AR to NOS in the endothelial cells of e-restricted-β3 mice (Suppl. Fig. 1D), in line with the established link between the vasodilatory properties of β3AR agonists and NO production [20].

### Cardiomyocyte-specific but not endothelial-specific β3AR expression confers cardioprotection upon selective β3AR agonist injection during ischemia–reperfusion

**Fig. 1:**
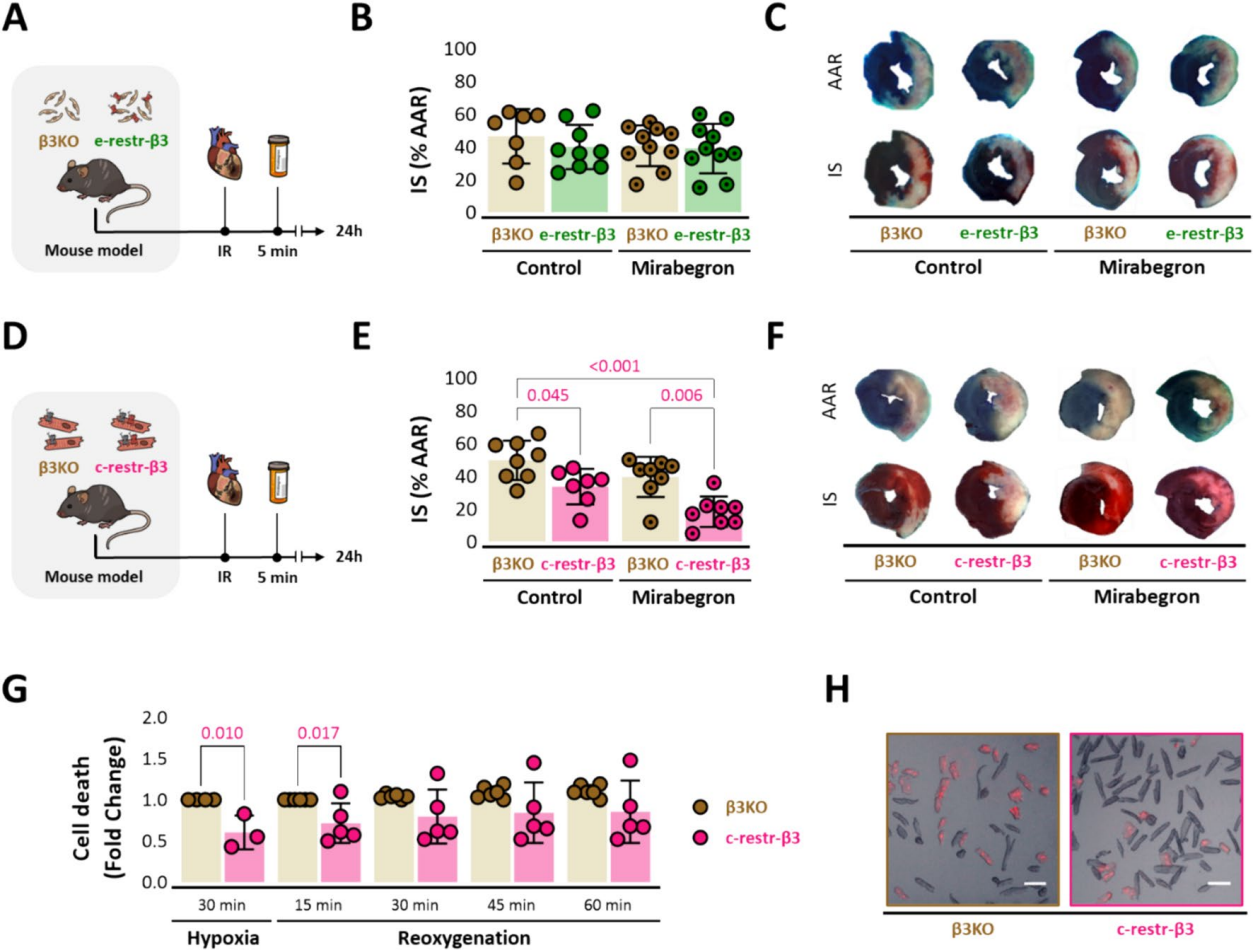
Cardiomyocyte-specific but not endothelium-specific expression of human β3AR confers cardioprotection upon selective β3AR agonist injection during ischemia–reperfusion. **A-F** Effect of endothelium-restricted versus cardiomyocyte-restricted β3AR activation on IR injury. **A** Experimental design and timeline. β3KO mice overexpressing the human β3AR in endothelial cells (e-restricted-β3) and their control littermates (β3KO) were subjected to left coronary artery occlusion for 45 min followed by reperfusion. Mice were randomized to receive the β3AR-specific agonist mirabegron (1 µg/kg) or vehicle by femoral-vein injection 5 min before reperfusion. Mice were sacrificed and heart samples collected 24 h after reperfusion. Histological quantification of IS determined by Evans Blue and TTC staining on LV slices. **B** e-restricted-β3 mice with mirabegron injection (*n* = 10) and without mirabegron (*n* = 9) versus β3KO littermate controls (*n* = 10 and *n* = 7). **C** Corresponding representative images of LV slices stained to reveal AAR (Evans Blue-negative) and extent of necrosis (TTC-negative area). **D** Experimental design and timeline of β3KO mice overexpressing the human β3AR in cardiomyocytes (c-restricted-β3) and their control littermates (β3KO) as described before. **E** c-restricted-β3 mice with mirabegron injection (*n* = 8) and without mirabegron (*n* = 7) versus β3KO littermate controls (*n* = 8 and *n* = 8, respectively). **F** Corresponding representative images of LV slices stained to reveal AAR (Evans Blue-negative) and extent of necrosis (TTC-negative area). **G** Cell death quantification of isolated adult mouse cardiac myocytes from β3KO mice (*n* = 4) and from c-restricted-β3 mice (*n* = 4) subjected to 30 min hypoxia (1% O_2_) and 1 h of reoxygenation. Cell death was assessed by propidium iodide (PI) internalization every 15  min after the beginning of reoxygenation. **H** Representative images of isolated cardiomyocytes after reoxygenation showing PI-negative rod-shaped fresh cardiomyocytes and PI-positive (red) dead cardiomyocytes from β3KO mice (left panel) and from c-restricted-β3 mice (right panel). Transgenic cells presented lower death rate. Scale bar, 100 µm. Data are presented as means ± SD and were analyzed by *t* test or one-way or two-way ANOVA. *p* values are indicated on graphs when significant. *β3AR* β3-adrenergic receptor, *IR* ischemia–reperfusion, *IS* infarct size, *LV* left ventricle, *TTC* triphenyltetrazolium chloride

Ischemia–reperfusion injury (IRI) was induced in e-restricted-β3 and c-restricted-β3 mice and control β3KO littermates by LAD occlusion for 45 min followed by reperfusion. Mice were randomized to receive a single bolus of the selective hβ3AR agonist mirabegron (1 µg/kg) or vehicle 5 min before reperfusion. LV samples were collected 24 h after reperfusion (Fig. 1A and D). RT-PCR analysis at baseline confirmed abundant expression of *ADBR3* mRNA in both e-restricted-β3 and c-restricted-β3 LV tissue (Suppl Fig. 2A-B).

In e-restricted-β3 mice, with endothelial-only hβ3AR expression, IS (calculated as the infarcted tissue-to-AAR ratio; see Methods) did not differ from β3KO controls (40 ± 14% vs 46 ± 17%, *p* = 0.313). Pre-reperfusion injection of the β3AR agonist mirabegron had no cardioprotective effect in e-restricted-β3 mice (IS = 39 ± 15% vs 41 ± 13% in β3KO controls, *p* = 0.726) (Fig. 1B). In contrast, in c-restricted-β3 mice, with cardiomyocyte-only hβ3AR expression, infarcts were significantly smaller than in β3KO controls (IS = 34 ± 11% vs 50 ± 12%, p = 0.045). Pre-reperfusion mirabegron injection further reduced IS in c-restricted-β3 mice (18 ± 9% vs 39 ± 12% in β3KO controls, *p* = 0.006) (Fig. 1E). Representative Evans Blue- and TTC-stained mid-ventricular cross-sectional slices of e-restricted-β3, c-restricted-β3, and β3KO hearts are presented in Fig. 1C and F. These results confirm that IS is reduced by exclusive stimulation of the β3AR in cardiomyocytes but not in endothelial cells.

Moreover, to confirm these results isolated cardiomyocytes were incubated 30 min under hypoxic conditions (1% O_2,_ pH = 6.8) to simulate in vivo ischemic conditions. Then cells were subjected to reoxygenation by addition of a normoxic buffer (pH = 7.4). The number of dead cells, quantified by propidium iodide internalization with a fluorescence microscope, was significantly lower in myocytes expressing the β3AR than in control myocytes after 30 min of hypoxia and at 15 after reoxygenation. We also observed lower cell-death at 30, 45 and 60 min of reoxygenation, although not significant (Fig. 1G). Representative images of platted cardiomyocytes at 60 min of reoxygenation are shown in Fig. 1H. This experiment further confirms the protective effect of β3AR stimulation in cardiac myocytes.

### β3-Adrenergic receptor overexpression in cardiomyocytes reduces infarct size and improves cardiac function after ischemia–reperfusion

**Fig. 2:**
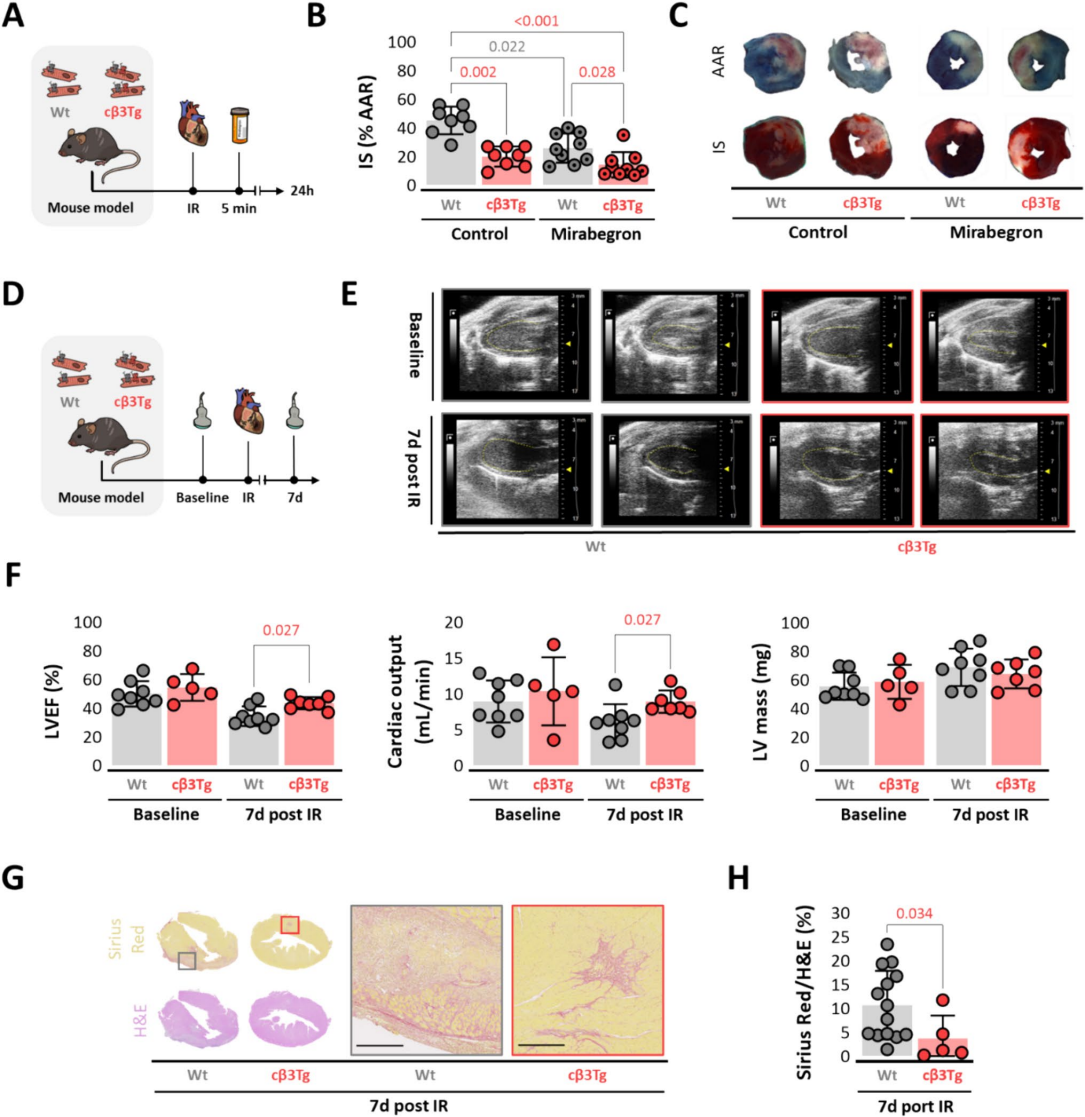
β3-adrenergic receptor overexpression in cardiomyocytes reduces infarct size and improves cardiac function after ischemia-reperfusion. **A-C** Effect of cardiomyocyte β3AR overexpression on IR injury in mice with intact endogenous β3AR expression. **A** Experimental design and timeline. Mice overexpressing the human β3AR in cardiomyocytes (cβ3Tg) and their control littermates (Wt) subjected to left coronary artery occlusion for 45 min followed by reperfusion. Mice were randomized to receive mirabegron (1 µg/kg) or vehicle by femoral-vein injection 5 min before reperfusion. Mice were sacrificed and heart samples collected 24 h after reperfusion. **B** Histological evaluation of IS in cβ3Tg mice with mirabegron injection (*n* = 9) and without mirabegron (*n* = 8) versus littermate Wt controls (*n* = 9 and *n* = 8). **C** Representative images of LV slices. **D-F** Echocardiography analysis of cardiac function after IR. **D** Experimental design and timeline. cβ3Tg mice and Wt littermate controls were examined by echocardiography at baseline and 7 d after IR. **E** Representative LV M-mode echocardiograms at baseline and 7 d after IR in cβ3Tg mice (*n* = 5 at baseline and *n* = 7 at 7 d) and Wt controls (*n* = 8 and *n* = 8). **F** Echocardiography assessment of LVEF, cardiac output, and LV mass in cβ3Tg and Wt mice at baseline and 7 d post IR. **G-H** Histological analysis of fibrosis in hearts of cβ3Tg and Wt mice 7 days after IR. Images show representative heart sections stained with hematoxylin–eosin (H&E) and sirius red. The chart shows fibrosis quantified as % of the fibrosis of Sirius red-stained area in cβ3Tg (*n* = 5) and Wt (*n* = 14) mice. Data are presented as means ± SD and were analyzed by *t* test or one-way or two-way ANOVA. *p* values are indicated on graphs when significant. *AAR* area at risk, *β3AR* β3-adrenergic receptor, *ADBR3* human β3-adrenergic receptor gene, *IR* ischemia–reperfusion, *IS* infarct size, *LV* left ventricle, *LVEF* left ventricular ejection fraction

Having established the effect of cardiomyocyte hβ3AR expression in isolation, we repeated the IRI procedure in cβ3Tg mice and Wt controls with intact endogenous mouse β3AR expression throughout the body **(**Fig. 2A). RT-PCR analysis at baseline confirmed abundant expression of *ADBR3* mRNA in cβ3Tg LV tissue (Suppl. Fig. 2C). Cardiomyocyte hβ3AR overexpression resulted in 50% smaller IS after IRI than Wt mice (20 ± 7% vs. 45 ± 10%, *p* = 0.002). Pre-reperfusion injection of mirabegron reduced IS both in cβ3Tg mice and in Wt mice, but infarcts were smaller in mirabegron-exposed cβ3Tg hearts than in their Wt counterparts (14% ± 8 vs. 26% ± 10%, *p* = 0.028) (Fig. 2B). Representative Evans Blue- and TTC-stained mid-ventricular cross-sections of cβ3Tg mice and Wt controls are shown in Fig. 2C.

To assess the impact of β3AR-mediated cardioprotection on cardiac systolic function, echocardiography was performed on new groups of cβ3Tg and Wt mice at baseline (before IRI) and again at 7 d after reperfusion (Fig. 2D). This analysis revealed better preservation of LVEF and cardiac output (CO) in cβ3Tg mice (Fig. 2E-F). This superior heart function was reflected in a significantly smaller fibrotic area at 7d post-reperfusion in in cβ3Tg mice, detected as the area ratio of Sirius-red to hematoxylin–eosin staining on heart sections (Fig. 2G-H).

### Cardiac β3-adrenergic receptor overexpression increases mitochondrial number by activating mitochondrial biogenesis

**Fig. 3:**
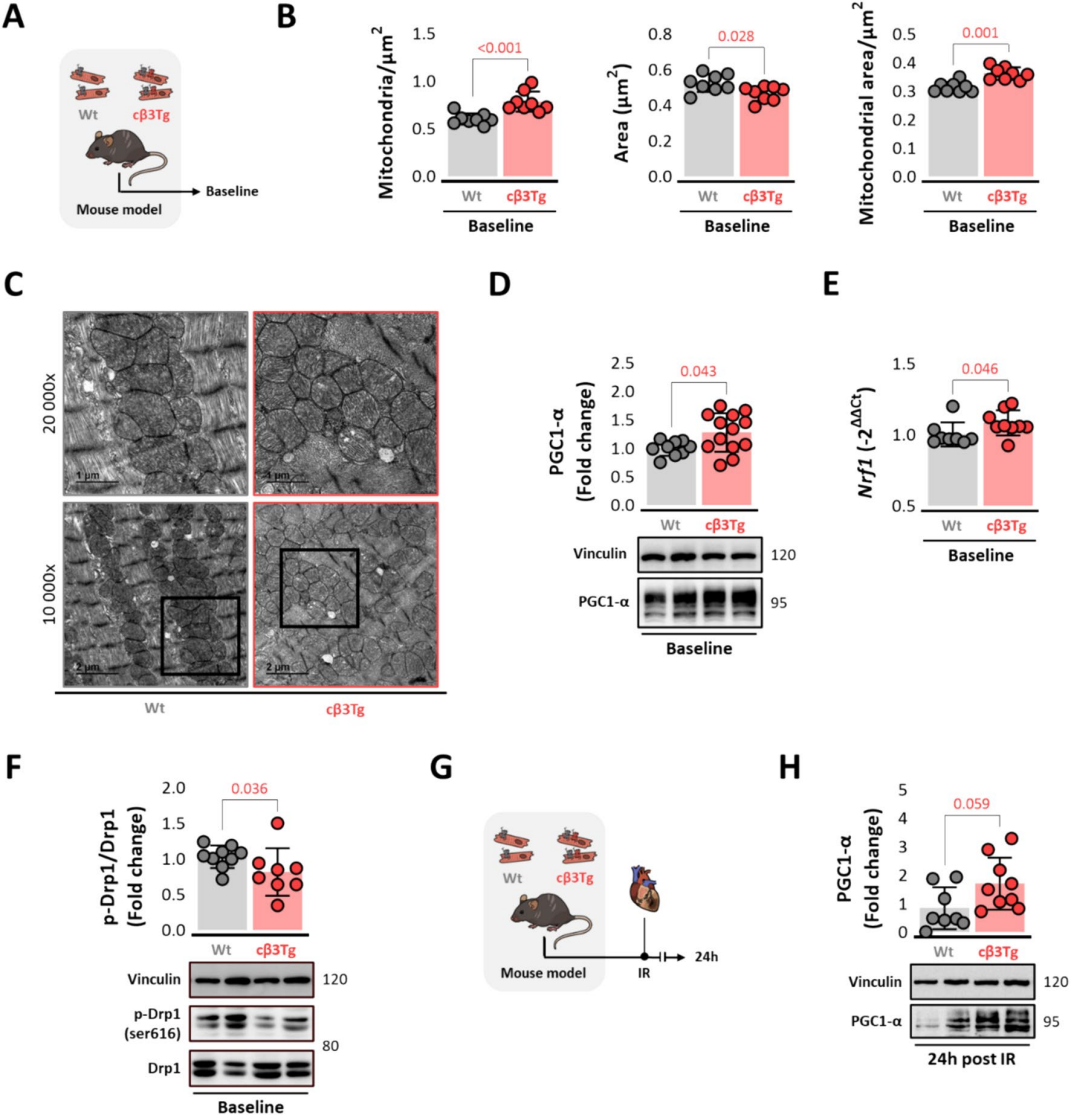
Cardiac β3-adrenergic receptor overexpression increases mitochondrial number by activating mitochondrial biogenesis. **A** Mice overexpressing the human β3AR in cardiomyocytes (cβ3Tg) and their control littermates (Wt) were sacrificed at baseline. **B-C** TEM analysis of LV sections from cβ3Tg mice (*n* = 8) and control Wt littermates (*n* = 8), revealing more abundant and smaller mitochondria in cβ3Tg hearts. Scale bars, 2 µm (top) and 1 µm (bottom). **D** LV western blot analysis of the mitochondrial biogenesis marker PGC1-α (Wt, *n* = 9; cβ3Tg, *n* = 13) and **E** LV RT-PCR analysis of the mitochondrial biogenesis regulator NRF1 (Wt, *n* = 8; cβ3Tg, *n* = 9) at baseline. **F** LV western blot analysis of Drp-1 mediated mitochondrial fission, showing lower Drp-1 phosphorylation on ser616 in cβ3Tg hearts (Wt, *n* = 9; cβ3Tg, *n* = 8) at baseline. **G** cβ3Tg mice and their control littermates (Wt) were subjected to left coronary artery occlusion for 45 min followed by reperfusion. **H** LV western blot analysis of PGC1-α (Wt, *n* = 8; cβ3Tg, *n* = 9) 24h after IR. *Drp-1* dynamin-related protein 1, IR, ischemia/reperfusion; LV, left ventricle; NRF1, nuclear respiratory factor 1; PGC1-α, peroxisome proliferator-activated receptor gamma coactivator 1-alpha; TEM, transmission electron microscopy

Given the key role of mitochondria in activating apoptosis, we next investigated the possible implication of the mitochondrial network in the mechanism of cardioprotection during IR in cβ3Tg mice. At baseline (no IRI exposure), cβ3Tg cardiomyocytes had significantly more abundant and smaller mitochondria than Wt controls but covering more cardiomyocyte area (Fig. 3A-C). Increased mitochondrial number was confirmed by increases in the mitochondrial content markers TOMM20 (Suppl. Fig. 5A-B) and citrate synthase (Suppl. Fig. 5C). The elevated mitochondrial content in cβ3Tg cardiomyocytes correlated with increased expression of the mitochondrial biogenesis protein PGC1-α (Fig. 3D) and upregulated mRNA expression of the downstream transcription factor NRF-1 (Fig. 3E). In line with these results, cβ3Tg cardiomyocytes contained fewer fragmented mitochondria than Wt cells, as indicated by lower serine 616 phosphorylation of the mitochondrial fission protein Drp-1 (Fig. 3F). No differences were observed in the mitochondrial fusion markers as mitofusins 1 and 2 (MFN1 and MFN2), OPA1, metalloendopeptidase OMA1, or Yme1L (Suppl. Fig. 7). To determine if this increase in mitochondrial biogenesis is maintained after IRI, these markers were analyzed in the left ventricle (LV) after 45 min of ischemia followed by 24 h of reperfusion (Fig. 3G). We found a strong trend of increased of PGC1-α after IR (Fig. 3H). Mitochondrial fragmentation, however, is not reduced any longer at these conditions (Suppl. Fig. 5D).

### Cardiac β3-adrenergic receptor overexpression uncouples the mitochondrial electron transport chain in homeostatic conditions to re-couple it upon reperfusion

**Fig. 4:**
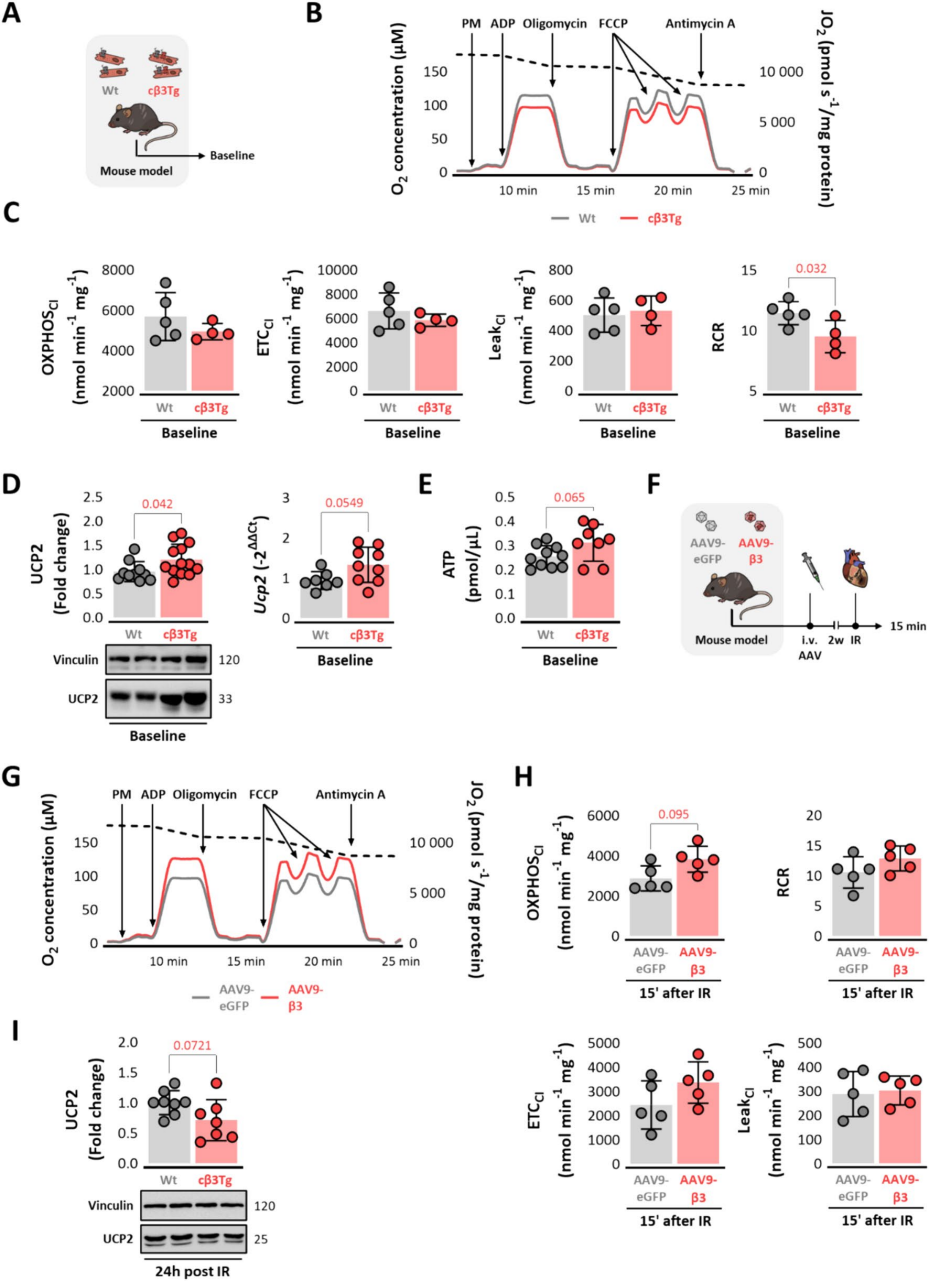
Cardiac β3-adrenergic receptor overexpression uncouples the mitochondrial electron transport chain at baseline and couple electron transport chain on early reperfusion. **A** Mice overexpressing the human β3AR in cardiomyocytes (cβ3Tg) and their control littermates (Wt) were sacrificed at baseline. **B** Representative respiration traces of isolated mitochondria from cβ3Tg (*n* = 5) and Wt (*n* = 4) mice. The discontinuous line indicates O_2_ concentration, and the gray (Wt) and red (cβ3Tg) lines indicate O_2_ flux per mg mitochondrial protein (JO_2_). Pyruvate and malate (PM) were added to support complex I (CI) flux in the routine state. This was followed by addition of ADP to drive oxidative phosphorylation (OXPHOS_CI_). Olygomycin was then added to inhibit complex V (Leak_CI_), and mitochondria were uncoupled by titration with the mitochondrial uncoupling agent FCCP to assess maximum electron transfer capacity (ETC_CI_). Residual non-mitochondrial oxygen consumption (ROX) was detected by addition of antimycin A to block electron transport. Arrows indicate the times of additions. **C** Mitochondrial respiration values for OXPHOS_CI_, ETC_CI_, Leak_CI_, and RCR. **D** LV western blot and RT-qPCR analysis of UCP2 protein levels (Wt, *n* = 10; cβ3Tg, *n* = 13) and *Ucp2* mRNA levels (Wt, *n* = 7; cβ3Tg, *n* = 9). **E** Baseline ATP cuantification in LV tissue. **F** Male Wt mice were transduced with AAV9-β3 or control AAV9-eGFP. At 2 weeks after infection, mice underwent left coronary artery occlusion for 45 min followed by reperfusion. Mice were sacrificed and heart samples collected 15 min after reperfusion. **G** Representative respiration traces of isolated mitochondria from AAV9-β3 (*n* = 5) and AAV9-*eGFP* (*n* = 5) mice. **H** Mitochondrial respiration values for OXPHOS_CI_, ETC_CI_, Leak_CI_, and RCR. (**I**) LV western blot of UCP2 (Wt, *n* = 8; cβ3Tg, *n* = 7) 24h after IRI. Data are presented as means ± SD and were analyzed by *t* test. *p* values are indicated on graphs when significant. Representative western blots are shown beneath graphs. *AAV* adeno-associated virus, *ADP* adenosine diphosphate, *ATP* adenosine triphosphate, *CI* complex I, *DAPI* 4′,6-diamidino-2-phenylindole, *Drp-1* dynamin-related protein 1, *FCCP* carbonyl cyanide-p-trifluoromethoxyphenylhydrazone, *LV* left ventricle, *NRF1* nuclear respiratory factor 1, *OXPHOS* oxidative phosphorylation, *PGC1-α* peroxisome proliferator-activated receptor gamma coactivator 1-alpha, *RCR* respiratory control ratio, *TEM* transmission electron microscopy, *TOMM20* translocase of outer mitochondrial membrane 20, *UCP2* mitochondrial uncoupling protein 2, *WGA* wheat germ agglutinin

Analysis of mitochondrial function showed that cβ3Tg heart mitochondrial homogenates responded normally to SUIT chemicals (Fig. 4A-B). Mitochondrial oxygen consumption in the OXPHOS_CI_, ETC_CI_, and Leak_CI_ states was similar in cβ3Tg and Wt mitochondria. However, analysis of the respiratory control ratio (RCR) revealed significantly impaired coupling efficiency in cβ3Tg cardiomyocytes (Fig. 4C) accompanied by a concomitant increase in UCP2 protein expression and mRNA levels (Fig. 4D). This impaired coupling on individual mitochondria does not affect the overall ATP production in the LV tissue (Fig. 4E). Overall mitochondrial coupling is also confirmed by elevated RCR in cβ3Tg isolated cardiomyocytes (Suppl. Fig. 6). We decided to analyze mitochondrial function in mice with AAV9-mediated β3AR overexpression mice subjected to 45 min of ischemia followed by 15 min of reperfusion **(**Fig. 4F). Mitochondrial homogenates responded normally to SUIT chemicals (Fig. 4G). Mitochondria from mice overexpressing β3AR show an increasing trend in oxygen consumption in the OXPHOS_CI_ and ETC_CI_ (Fig. 4H), and more importantly, coupling efficiency is restored (i.e. not impaired like in homeostatic (baseline) conditions) (Fig. 4H). Although both models exhibit the same patterns following overexpression, we cannot completely rule out differences between animals with transgenic overexpression and those treated with AAV vectors. Levels in UCP2 protein expression show a trend to downregulation after 24h of IR, in line with mitochondrial coupling on early reperfusion (Fig. 4I). These results confirm that mitochondrial network´s preconditioning driven by β3AR overexpression allow individual mitochondria to better tolerate the ischemic insult and confer them greater responsiveness upon reperfusion.

### Cardiac β3-adrenergic receptor overexpression upregulates markers of cell survival in homeostatic conditions and promotes antioxidant response after ischemia/reperfusion injury

**Fig. 5:**
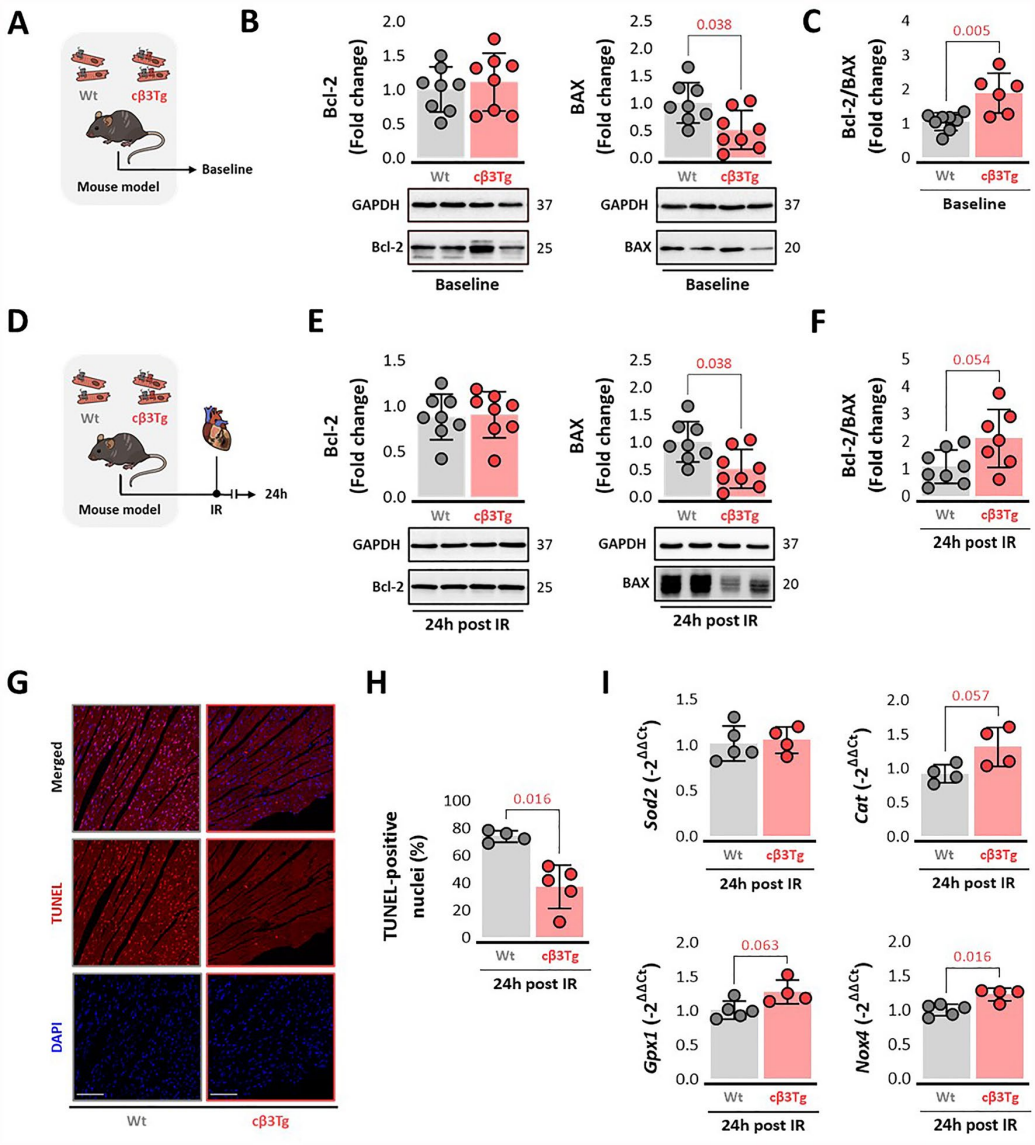
Cardiac β3-adrenergic receptor overexpression downregulates markers of cell survival and promotes antioxidant response after ischemia-reperfusion injury. **A** Mice overexpressing the human β3AR in cardiomyocytes (cβ3Tg) and their control littermates (Wt) were sacrificed at baseline. **B**-**C** LV western blot analysis of the apoptosis-related proteins BAX and Bcl-2 and the cell-death switch represented as the Bcl-2/BAX ratio (Wt, *n* = 8; cβ3Tg, *n* = 8). **D** cβ3Tg mice and their control littermates (Wt) were subjected to left coronary artery occlusion for 45 min followed by reperfusion. **E**-**F** LV western blot analysis of BAX and Bcl-2 and the cell-death switch represented as the Bcl-2/BAX ratio (Wt, *n* = 8; cβ3Tg, *n* = 8) 24 h after IRI. **G-H** Representative heart confocal images and ratio of nuclei stained by TUNEL assay compared to total nuclei (Wt, *n* = 4; cβ3Tg, *n* = 5) 24h after IR **I**. RT-qPCR analysis of antioxidant genes *Sod2, Cat, Gpx1* and *Nox4* (Wt, *n* = 8; cβ3Tg, *n* = 8) 24 h after IR. Data are presented as means ± SD and were analyzed by *t* test. *p* values are indicated on graphs when significant. Representative western blots are shown beneath graphs. *BAX* bcl-2-like protein 4, *Bcl-2* B-cell lymphoma 2, *IR* ischemia–reperfusion, *LV* left ventricle, *Sod2* superoxide dismutase 2 gene, *Cat* catalase gene, *Gpx1* glutathione peroxidase 1 gene, *Nox4* NADPH oxidase 4, *TUNEL* terminal deoxynucleotidyl transferase dUTP nick end labeling, *DAPI* 4′,6-diamidino-2-phenylindole

An analysis of the baseline expression of mitochondrial quality control (QC) markers identified relatively lower expression of the pro-apoptosis protein BAX in cβ3Tg hearts, with the anti-apoptosis regulator Bcl-2 showing no difference between genotypes (Fig. 5A-B). The Bcl-2/BAX ratio, an index of susceptibility to apoptotic cell death (low Bcl-2/Bax ratio represents cell death susceptibility, while a higher ratio suggest resistance to apoptotic stimuli) [32, 66], was thus diminished in Wt control hearts compared to cβ3Tg hearts (Fig. 5C). To measure cellular response after IRI, these markers were analyzed in the left ventricle (LV) after 45 min of ischemia followed by 24 h of reperfusion (Fig. 4D). Pro-apoptosis protein BAX and anti-apoptotic regulator Bcl-2 maintain the same differences as baseline cβ3Tg hearts (Fig. 5E-F). Thus, cardiomyocyte survival is promoted in β3AR overexpression confirmed by greater Bcl-2/BAX ratio after IR (Fig. 5F). TUNEL staining results show less TUNEL-positive nuclei, marker of DNA damage, in cβ3Tg AAR heart sections compared to control littermates (Fig. 5G-H). Finally, RT-qPCR analysis show an antioxidant response by expressing genes of Catalase, Gpx1 and Nox4 after IR (Fig. 5I). These results show that β3AR overexpression in cardiomyocytes improve cell survival and promotes antioxidant response after IR.

### Cardiac β3-adrenergic receptor overexpression downregulates mitophagy markers in homeostatic conditions to restore them upon reperfusion

**Fig. 6:**
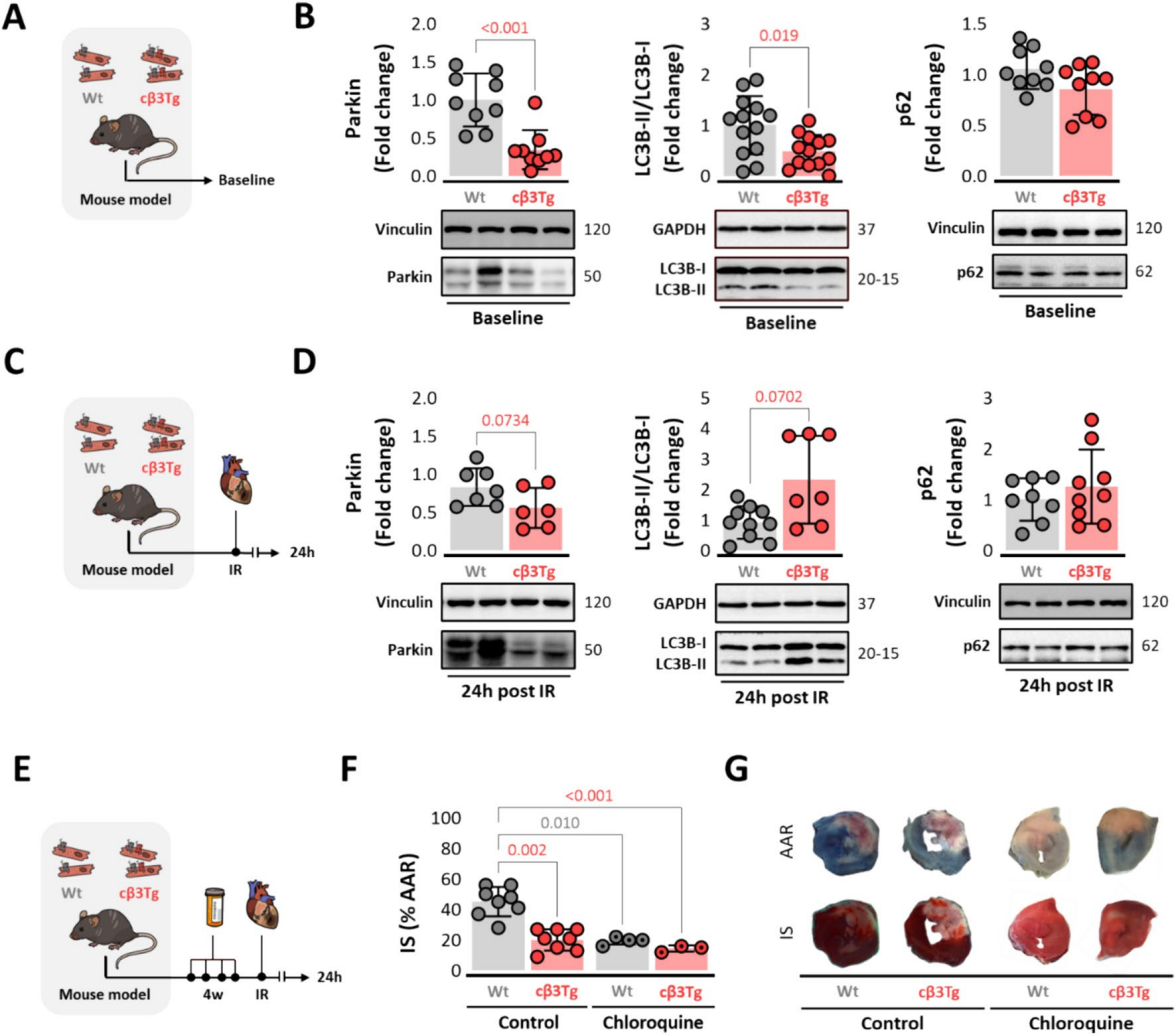
Cardiac β3-adrenergic receptor overexpression downregulates markers of mitophagy at baseline and triggers general autophagy after ischemia/reperfusion. **A** Mice overexpressing the human β3AR in cardiomyocytes (cβ3Tg) and their control littermates (Wt) were sacrificed at baseline. **B** Baseline LV western blot analysis of mitophagy markers: parkin, LC3B-II/LC3B-I ratio and p62 (Wt, *n* = 10–13; cβ3Tg, *n* = 10–13). **C** cβ3Tg mice and their control littermates (Wt) were subjected to left coronary artery occlusion for 45 min followed by reperfusion. **D** LV western blot analysis of parkin, LC3B-II/LC3B-I ratio and p62 (Wt, *n* = 7–10; cβ3Tg, *n* = 6–9) 24 h after IR. **E** Experimental design and timeline. cβ3Tg mice and control littermates were randomized to receive a daily oral dose of 1.15–2.02 mg chloroquine or placebo for 4 weeks before left coronary artery occlusion for 45 min followed by reperfusion. Mice were sacrificed and heart samples collected 24 h after reperfusion. **F** Histological evaluation of IS determined by Evans Blue and TTC staining on LV slices in cβ3Tg mice with chloroquine injection (*n* = 3) and without chloroquine (*n* = 8) versus littermate Wt controls (*n* = 4 and *n* = 8). **G** Representative images of LV slices. Data are presented as means ± SD and were analyzed by *t* test and one-way ANOVA. *p* values are indicated on graphs when significant. Representative western blots are shown beneath graphs. *IR* ischemia–reperfusion, *IS* infarct size, *LC3B* microtubule associated protein 1 light chain 3 beta, *LV* left ventricle, *p62* ubiquitin-binding protein p62, Parkin, parkin RBR E3 ubiquitin protein ligase

Mitochondria with a weakened membrane potential fail to clear PINK1 from the outer to the inner membrane for cleavage by PARL, and the resulting accumulation of full-length PINK1 on the outer membrane recruits parkin, which targets the damaged mitochondria for degradation through selective autophagy (mitophagy) [53]. Mitophagy was inhibited in cβ3Tg hearts, as indicated by parkin down expression (Fig. 6A-B). cβ3Tg hearts also showed a downregulation of the membrane-bound LC3B-II isoform, indicating a downregulation in autophagosome assembly in homeostatic conditions (Fig. 6B). The downregulated autophagy could not be explained by a defect in autophagic flux since there were no between-genotype differences in the content of ubiquitin-binding protein p62 or Beclin-1 mRNA and protein expression (Fig. 6B and Suppl. Fig. 8A-B). After 45 min of ischemia followed by 24 h of reperfusion, a non-significant trend towards reduced parkin protein levels was found (Fig. 6E-D). However, LC3B-II isoform ratio is no longer downregulated upon reperfusion (Fig. 6D), suggesting its restoration under these conditions. No autophagic flux disruptions were observed after IR in p62 or Beclin-1 mRNA and protein expression (Fig. 6D and Suppl. Fig. 8C).

To confirm the beneficial impact of autophagy downregulation on IRI, we treated cβ3Tg mice and Wt littermates with a daily oral dose of the autophagic flux inhibitor chloroquine for 4 weeks before the IRI procedure (Fig. 6E). In Wt animals, chloroquine treatment reduced IS (19 ± 2% vs 45 ± 10% in chloroquine- and vehicle-treated Wt mice, respectively, *p* = 0.010). Conversely, in cβ3Tg mice, with established autophagy downregulation, chloroquine had no further cardioprotective effect (20 ± 7% vs 14 ± 2% in chloroquine- and vehicle-treated cβ3Tg mice, respectively, *p* = 0.315) (Fig. 6F-G). Autophagic flux was shown to be preserved after acute intraperitoneal treatment with leupeptin and chloroquine in LC3B-II and p62 protein levels (Suppl Fig. 9). These findings suggest that the steady-state inhibition of mitophagy in cβ3Tg mice mediates IS limitation after IRI in these animals.

### Gene therapy by AAV-mediated cardiac β3-adrenergic receptor overexpression reduces infarct size in mice

Having demonstrated that constitutive cardiomyocyte overexpression of human β3AR limits IS after IR, we next explored gene therapy as a more translational strategy to overexpress β3AR. We designed an AAV transfer plasmid encoding human *ADRB3* under the control of the troponin T promoter to generate an AAV9 virus directing cardiomyocyte-specific hβ3AR expression (AAV9-β3). An AAV transfer plasmid encoding *eGFP* was used as a control [47], and AAV9-β3 or control AAV9-eGFP were used to transduce 8 week-old Wt mice.

**Fig. 7:**
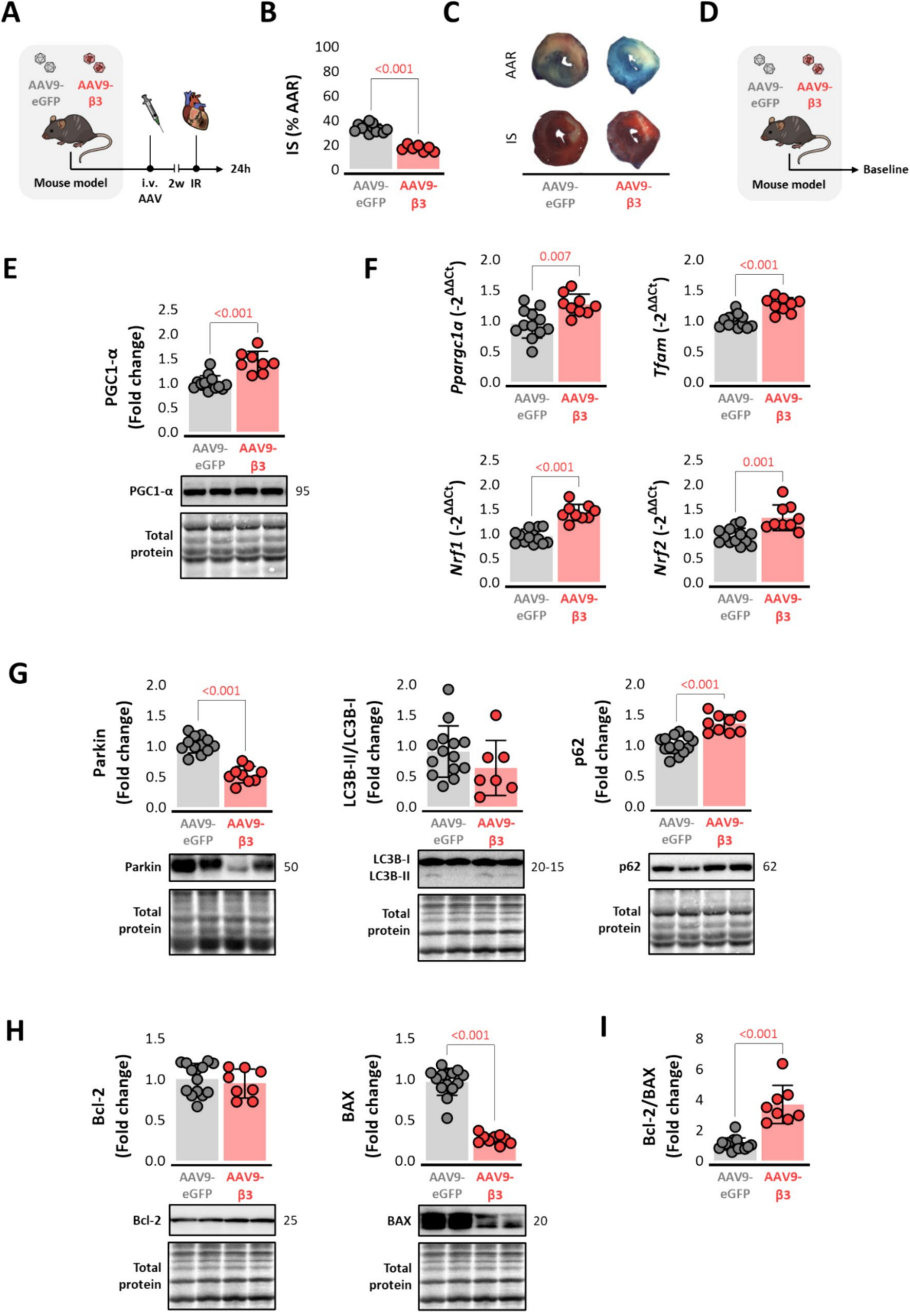
AAV-mediated cardiac β3-adrenergic receptor gene therapy reduces infarct size. Experimental design and timeline. **A** Male Wt mice were transduced with AAV9-β3 (red) or control AAV9-eGFP (gray). At 2 weeks after infection, mice underwent left coronary artery occlusion for 45 min followed by reperfusion. Mice were sacrificed and heart samples collected 24h after reperfusion. **B** Histological evaluation of IS determined by Evans Blue and TTC staining on LV slices in AAV9-β3 mice (*n* = 7) versus AAV9-eGFP controls (*n* = 12). **C** Representative images of LV slices. **D** Mice transduced with AAV9-β3 and control AAV9-eGFP were sacrificed at baseline. **E-I** LV western blot and RT-qPCR analysis of mitochondrial quality control-related proteins in AAV9-β3 mice (*n* = 14) and AAV9-eGFP controls (*n* = 9): **E** mitochondrial biogenesis marker PGC1-α; **F** mitochondrial biogenesis genes *Ppargc1a, Tfam, Nrf1* and *Nrf2;* **G** mitophagy-related proteins parkin and p62; **H** the apoptosis-related proteins BAX and Bcl-2. **I** Cell-death switch represented as the BAX/Bcl-2 signal intensity ratio. Data are presented as means ± SD and were analyzed by *t* test. *p* values are indicated on graphs when significant. Representative western blots are shown beneath graphs. *AAR* area at risk, *AAV* adeno-associated virus, *BAX* bcl-2-like protein 4, *Bcl-2* B-cell lymphoma, *β3AR* β3-adrenergic receptor, *ADBR3* human β3AR gene, *IR* ischemia–reperfusion, *IS* infarct size, *LC3B* microtubule associated protein 1 light chain 3 beta, *LV* left ventricle, *p62* ubiquitin-binding protein p62, *Parkin* parkin RBR E3 ubiquitin protein ligase, *PGC1-α* peroxisome proliferator-activated receptor gamma coactivator 1-alpha, *Ppargc1a* PGC1-α gene, *Tfam* mitochondrial transcription factor A gene, *Nrf1* nuclear respiration factor 1 gene, *Nrf2* nuclear respiration factor 2 gene

Two weeks after AAV infection, mice underwent the IRI procedure (Fig. 7A) after confirmation of correct gene delivery by RT-PCR (Suppl Fig. 2D). Mice with AAV-mediated hβ3AR overexpression in cardiomyocytes had significantly smaller infarcts than controls (IS = 17 ± 3% vs. 34 ± 3% of AAR, *p* < 0.0001) (Fig. 7B-C). Analysis of baseline (no IRI) (Fig. 7D) expression of mitochondrial QC and mitochondrial biogenesis markers in AAV-infected mice revealed similar alterations to those detected in transgenic mice with constitutive cardiomyocyte hβ3AR overexpression (Fig. 5E-I and Supp. Info 7D-E).

## Discussion

This study provides evidence that the cardioprotective effect of pre-reperfusion β3AR activation is mediated by cardiomyocytes and not endothelial cells. Healthy transgenic mice overexpressing human β3AR in cardiomyocytes displayed a remodeling of the mitochondrial network in these cells, including upregulation of mitochondrial biogenesis and downregulation of mitophagy, fission, and autophagy. We decided to use the human *ADBR3* gene instead of the murine one, first of all for its clear differentiation with the endogenous murine gene *Adbr3* in the β3KO mice models, and finally due to its translational implications. We propose that this close relationship between constitutive β3AR activation and cardiomyocyte mitochondrial dynamics renders these organelles more resistant to IRI. Our results also demonstrate the cardioprotective effect of AAV-mediated cardiomyocyte-specific β3AR overexpression, establishing the feasibility of a gene-therapy strategy for AMI based on such an approach.

In a previous study, we linked the cardioprotective effect of human β3AR activation in mice to activation of the NO–cGMP pathway [47]. Since β3AR activation can increase NO production in both cardiomyocytes [5, 78] and coronary endothelial cells [20], both cell types have been suggested as mediators of the cardioprotective effect of β3AR stimulation during IRI [36]. Other studies have also pointed out that factors excreted from endothelial cells can modulate cardiomyocyte contraction [69]. Also, coronary vasculature plays a role in reperfusion injury, as gentle reperfusion has been shown to be cardioprotective [34, 37, 55]. In our study, transgenic mice overexpressing β3AR only in the endothelium showed no protection against myocardial IRI, even when the selective β3AR agonist mirabegron was injected before reperfusion. The in vitro myography experiments confirmed the specificity of mirabegron-stimulated human β3AR activation in endothelial cells, excluding the possibility of a non-functional receptor or a lack of an effect of mirabegron. This study, thus, provides the first evidence that β3AR activation in endothelial cells is not responsible for the cardioprotective action of β3AR stimulation against IRI. β3AR stimulation in the endothelium has been shown to induce vasodilation [19, 50]. Thus, c-restricted-β3 mice (lacking β3AR in endothelium) might display a vasoconstrictive phenotype. Vasoconstriction immediately after ischemia-reperfusion can induce a “gentle reperfusion” phenomenon and paradoxically be cardioprotective [55]. While this could be an alternative cardiomyocyte-independent mechanism of protection in c-restricted-β3 mice that could partially explain the infarct size reduction, its evaluation is beyond the scope of this work.

In contrast, strong protection against IRI was observed in mice with hβ3AR overexpression in cardiomyocytes, and this cardioprotective effect was enhanced by pre-reperfusion stimulation with mirabegron. These results are in line with previous in vitro and in vivo studies showing a protective role of the β3AR in cardiomyocytes [5, 35].

The study of the cell compartment responsible for the protection afforded by a pharmacological agent has translational implications. Another βAR modulating agent, metoprolol (a β1AR selective antagonist) has been shown to protect from myocardial ischemia-reperfusion injury by an affect driven to a big extent by neutrophils [14, 49]. Thanks to the discovery of this extra cardiac mechanism of cardioprotection, this intervention has been tested in other pathological conditions such as acute respiratory distress syndrome [15]. It is fair to acknowledge that metoprolol has not been shown to exert cardioprotection in other myocardial infarction models, such as Gottingen minipigs [45]. The variable effect of βAR modulating agents in different animal strains can be explained by the genetic background of different animals making them more responsive to βAR modulation [41].

For our investigation of the mechanism of β3AR-mediated cardioprotection, we focused on the mitochondrial network because this is known to play a critical role in IRI [40, 46]. Moreover, previous work in our laboratory supports a direct protective action of β3AR stimulation in cardiomyocytes through delayed opening of the mitochondria permeability transition pore [27]. In the present study, we found that β3AR overexpression in cardiomyocytes is associated with enhanced mitochondrial biogenesis, resulting in higher numbers of mitochondria that are smaller than those in cells from Wt hearts. Active mitochondrial biogenesis was further supported by the upregulation of the mitochondrial biogenesis markers PGC1-α and NFR1. The generation of new mitochondria can improve mitochondrial function and has been described in adipocytes after β3AR activation [4] and also in cerebral ischemia after activation of PGC-1α [80]. In adipocytes, β3AR-mediated PGC1-α activation is implicated in heat generation and energy expenditure by uncoupling oxidative phosphorylation through UCP1 in adipocytes [4, 61]. In cβ3Tg mice, upregulated cardiomyocyte expression of the UCP1 homolog UCP2 may similarly be triggered by PGC1-α activation. Uncoupling of the mitochondrial ETC in cβ3Tg cardiomyocytes was confirmed by the low RCR detected in the respirometry analysis. UCP2-mediated ETC uncoupling has been shown to have a cardioprotective effect in the context of IRI by reducing ROS production [75, 79]. We propose that increased mitochondrial abundance and ETC uncoupling upon PGC1-α activation preconditions cβ3Tg cardiomyocytes to better withstand oxidative stress during IRI. UCP2 mediated uncoupling of the ETC results in less ATP production per individual mitochondrial [62]. This under-energetic state triggered by β3AR could activate AMPK signaling pathway and activate PGC1-α [13], resulting in fresh mitochondria generation and compensating uncoupling stage by an increase in mitochondrial mass. This context would provide more ATP in a less oxidative environment, preconditioning the heart to better tolerate IRI. This alternative hypothesis can be fed back with UCP2 activation by PGC1-α.

Mitochondrial turnover is determined by a finely regulated balance between biogenesis and removal through mitophagy and general autophagy mechanisms [60]. Although mitophagy is an important mechanism for preventing the accumulation of dysfunctional mitochondria, there is evidence that mitophagy can be harmful during IRI [77]. For example, parkin-mediated mitophagy has deleterious effects in reperfused mouse hearts by opening the mPTP and inducing excessive mitochondrial elimination [71, 82]. In healthy conditions, cβ3Tg hearts showed a reduction in the key mitophagy mediator parkin, paralleled by low levels of the general autophagy marker LC3B-II [64]. However, the unaltered expression of other autophagosome formation proteins p62 and Beclin-1 suggests that β3AR overexpression does not block baseline autophagic flux [23]. We confirmed the benefits of autophagy reduction during IRI by treating mice with the antimalarial drug chloroquine, which blocks lysosomal degradation and inhibits the final stage of autophagy [17]. While chloroquine reduced IS in Wt mice, the failure of this treatment to provide further benefit against IRI in cβ3Tg mice strongly suggests that the cardioprotection provided by cardiac β3AR overexpression involves mitophagy inhibition in combination with enhanced mitochondrial biogenesis during homeostatic conditions. It is known that autophagy serves as a crucial protective mechanism by removing damaged cellular components upon different injuries, including upon reperfusion [38]. Here we observed an inhibition of quality control (mainly mitophagy) in homeostatic conditions, something that might be perceived as counterintuitive given the protection observed in these mice. However, after IRI, we observed a restoration in the quality control markers, with an increase of the general autophagic marker LC3B-II. This opposite quality control status (reduced in homeostatic conditions and restored after ischemia-reperfusion) can explain the capacity of cardiomyocytes with overexpression of β3AR to cope with acute insults with a massive quality control reserve. However, these changes in the general autophagic markers are mild and could be misunderstood depending on the time and activity of the autophagic flux. Further studies are needed to clarify autophagic response after β3AR overexpression-mediated reduction of mitophagy.

Finally, after IRI, mice overexpressing β3AR in cardiomyocyte trigger a high antioxidant response to better tolerate the insult, confirmed by the higher mRNA levels of *Cat* and *Gpx1* genes [33, 56]. *Nox4* gene expression is also known to modulate redox signaling by an expression of the antioxidant genes in cardiomyocytes [8], thus further confirmation of the antioxidant response in B3AR transgenic mice.

Cardiomyocytes from cβ3Tg mice also showed evidence of a baseline downregulation of apoptosis, a process closely connected to autophagy [74]. Canonical mitochondria-induced apoptosis is mainly regulated by pro-apoptotic proteins like BAX [68] and it is the main contributor to cardiomyocyte loss during IRI [77]. which was downregulated in cβ3Tg hearts. After IR this downregulation is maintained, promoting the cell survival during ischemia-reperfusion. Moreover, the downregulated activity of the apoptotic mitochondrial fission marker Drp-1 indicates not only inhibition of mitochondrial fission in cβ3Tg hearts but also delays to apoptosis onset and cell death, which have been demonstrated to have cardioprotective effects [22, 81].

The ability to recapitulate cardioprotection against IRI with an AAV virus directing cardiac-specific hβ3AR expression establishes proof-of-principle of the therapeutic potential of this strategy for secondary prevention. Transduction of AAV9-β3 not only resulted in smaller infarcts induced 2 weeks after infection, but also induced bigger alterations in mitochondrial QC markers to those triggered by constitutive transgenic cardiomyocyte hβ3AR overexpression. Bigger differences can be explained as an acute signaling activation compared to chronic signaling pathways observed in transgenic mice. These results thus confirm that AAV-mediated cardiac-specific β3AR overexpression is a suitable gene-therapy for preconditioning the mitochondrial network to tolerate IRI. Other interventions that enhance β3AR expression in the heart include exercise [10] and treatment with the beta-blocker metoprolol [12, 67, 76]. AAV-driven β3AR overexpression could provide baseline protection in patients with a history of myocardial infarction, as well as boosting the benefit of pre-reperfusion treatment with β3AR agonists in these high-risk patients.

Our study demonstrates that the cardioprotection provided by β3AR signaling is mediated by expression in cardiomyocytes, and not the endothelium. β3AR overexpression in cardiomyocytes results in a downregulation of mitochondrial quality control mechanisms (mitophagy and general autophagy) together with hyperactivation of mitochondrial biogenesis. These effects result in a remodeled mitochondrial network characterized by an increased number of small mitochondria less prone to ROS production. Together, these data suggest that mitochondria in cardiomyocytes overexpressing the β3AR are preconditioned to better tolerate IRI. Altogether, these results would explain the cardioprotective effect of β3AR agonist upon IRI and highlight that promoting cardiac β3AR expression either naturally or artificially stands as relevant strategy to reduce consequences of an acute myocardial infarct.

## Supplementary Information

Below is the link to the electronic supplementary material.

Supplementary file1 (JPG 194 KB)

Supplementary file2 (JPG 57 KB)

Supplementary file3 (JPG 157 KB)

Supplementary file4 (JPG 137 KB)

Supplementary file5 (JPG 219 KB)

Supplementary file6 (JPG 105 KB)

Supplementary file7 (JPG 172 KB)

Supplementary file8 (JPG 234 KB)

Supplementary file9 (JPG 152 KB)

Supplementary file10 (JPG 529 KB)

Supplementary file11 (JPG 288 KB)

Supplementary file12 (JPG 398 KB)

Supplementary file13 (JPG 465 KB)

Supplementary file14 (JPG 556 KB)

Supplementary file15 (JPG 554 KB)

Supplementary file16 (JPG 476 KB)

Supplementary file17 (JPG 409 KB)

Supplementary file18 (JPG 392 KB)

Supplementary file19 (DOCX 18 KB)

## Data Availability

Data are available from the corresponding author upon reasonable request.
