# Supplementary figures and images for: β3-Adrenergic receptor overexpression in cardiomyocytes preconditions mitochondria to withstand ischemia–reperfusion injury
Source: Basic Res Cardiol. 2024 Aug 12;119(5):773–94. doi: 10.1007/s00395-024-01072-y (PMC11461581; doi:10.1007/s00395-024-01072-y)

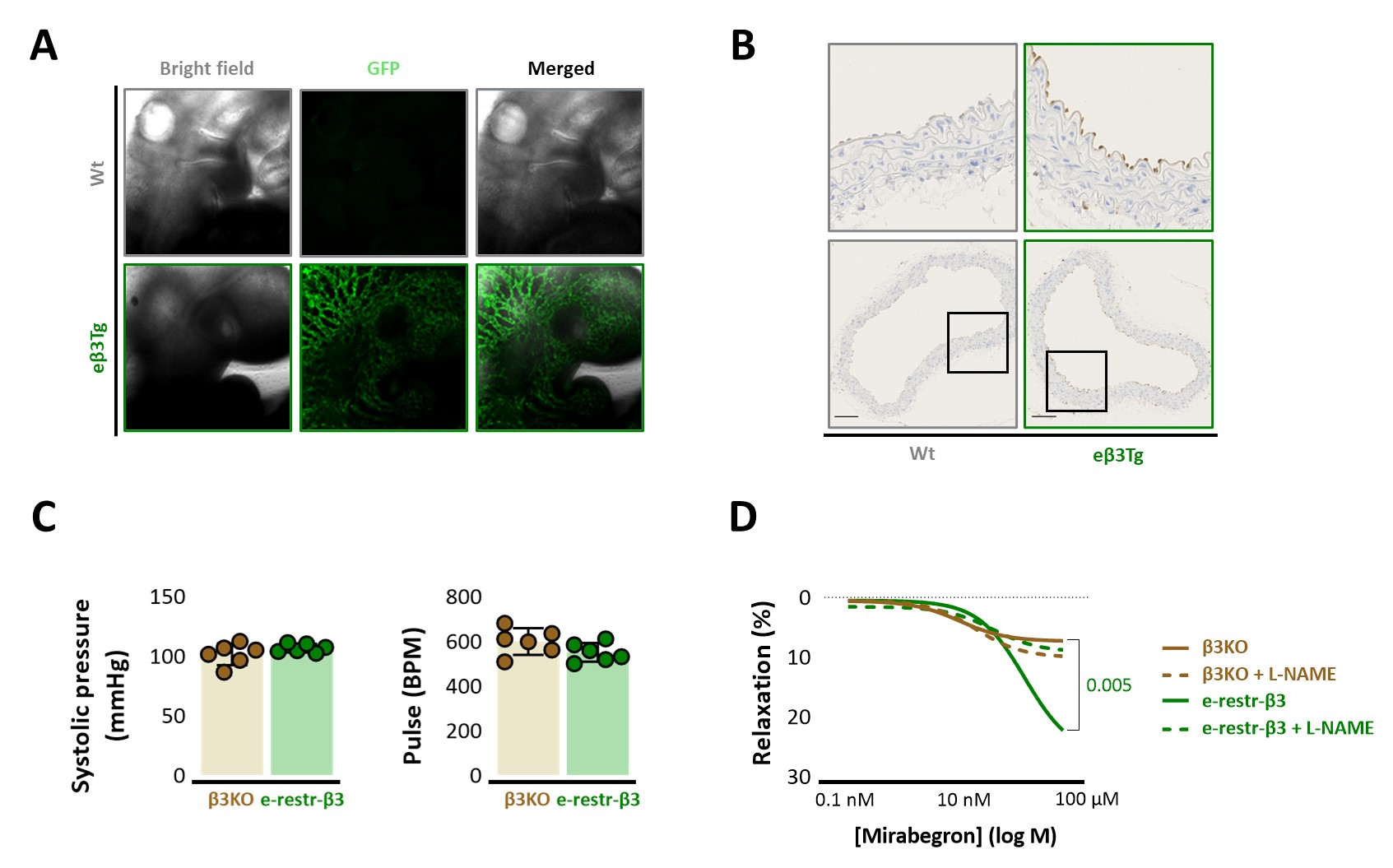

Supplement: Supplementary file 1 — Supplementary file1 (JPG 194 KB) [file 395_2024_1072_MOESM1_ESM.jpg]

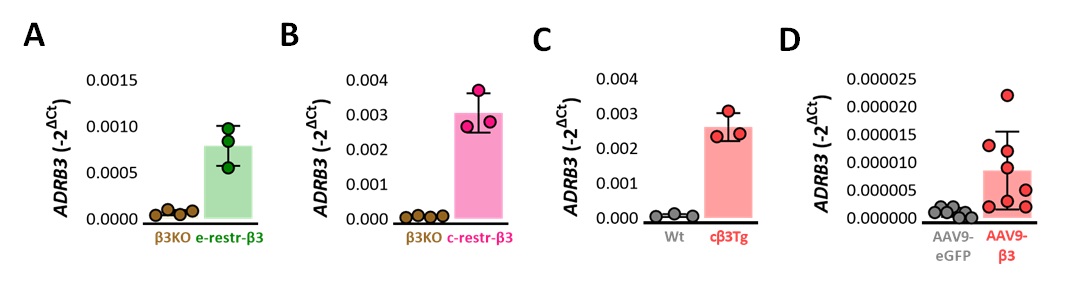

Supplement: Supplementary file 2 — Supplementary file2 (JPG 57 KB) [file 395_2024_1072_MOESM2_ESM.jpg]

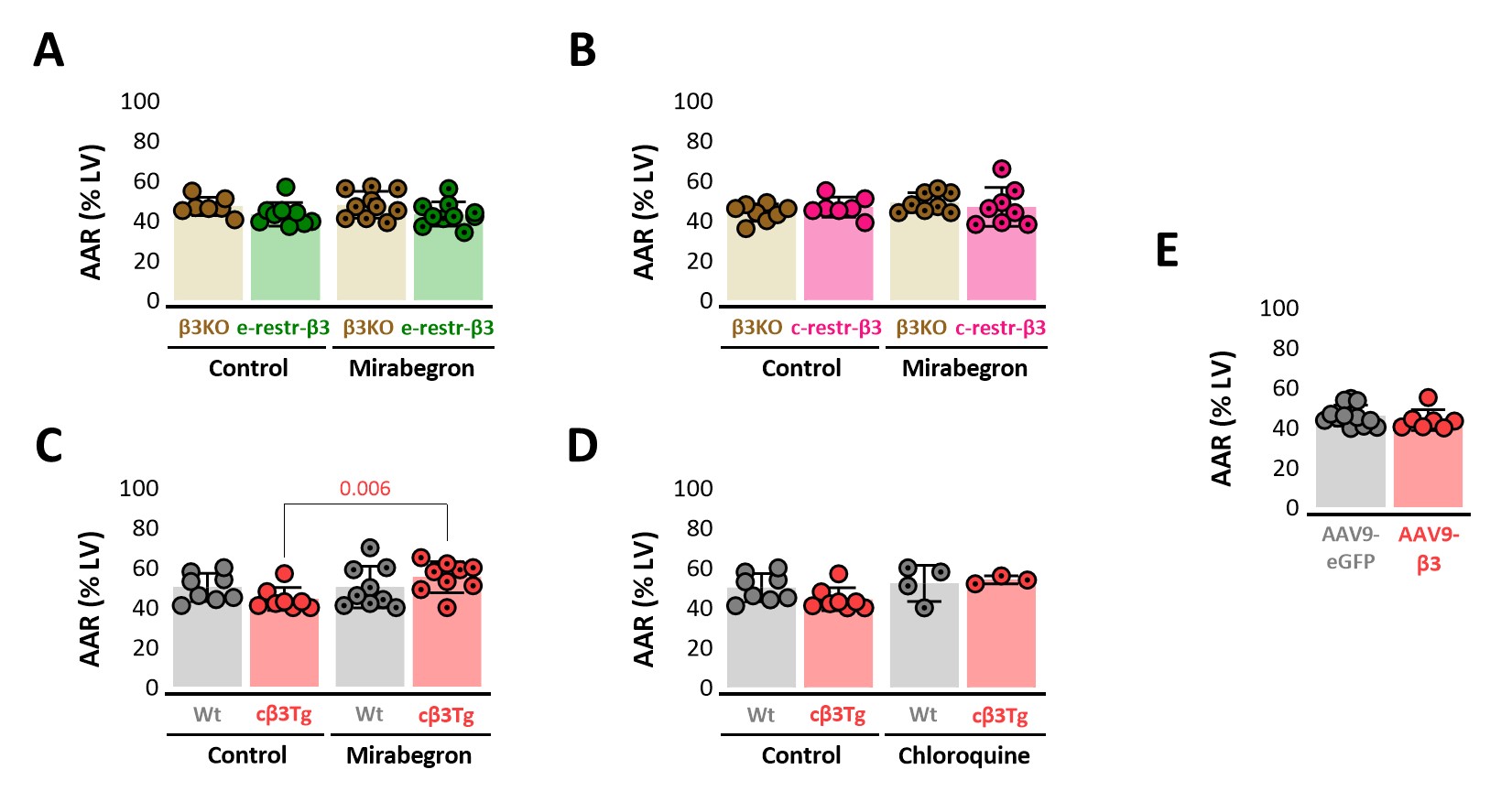

Supplement: Supplementary file 3 — Supplementary file3 (JPG 157 KB) [file 395_2024_1072_MOESM3_ESM.jpg]

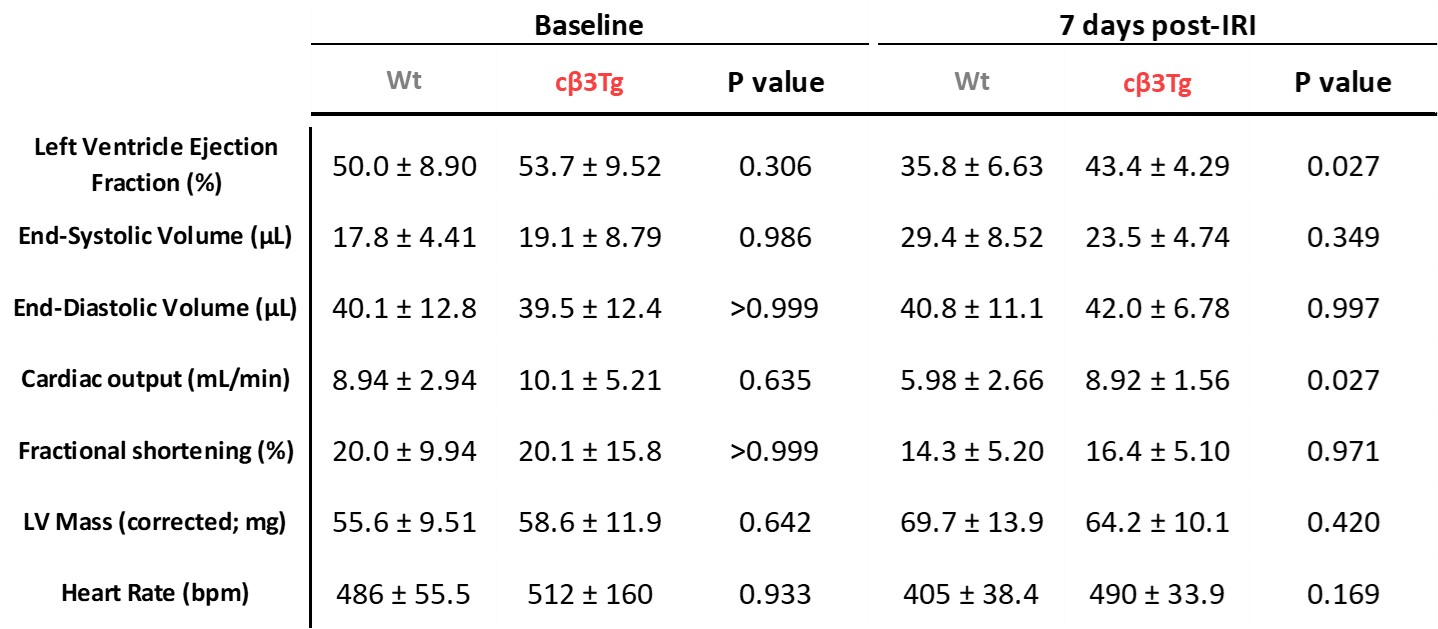

Supplement: Supplementary file 4 — Supplementary file4 (JPG 137 KB) [file 395_2024_1072_MOESM4_ESM.jpg]

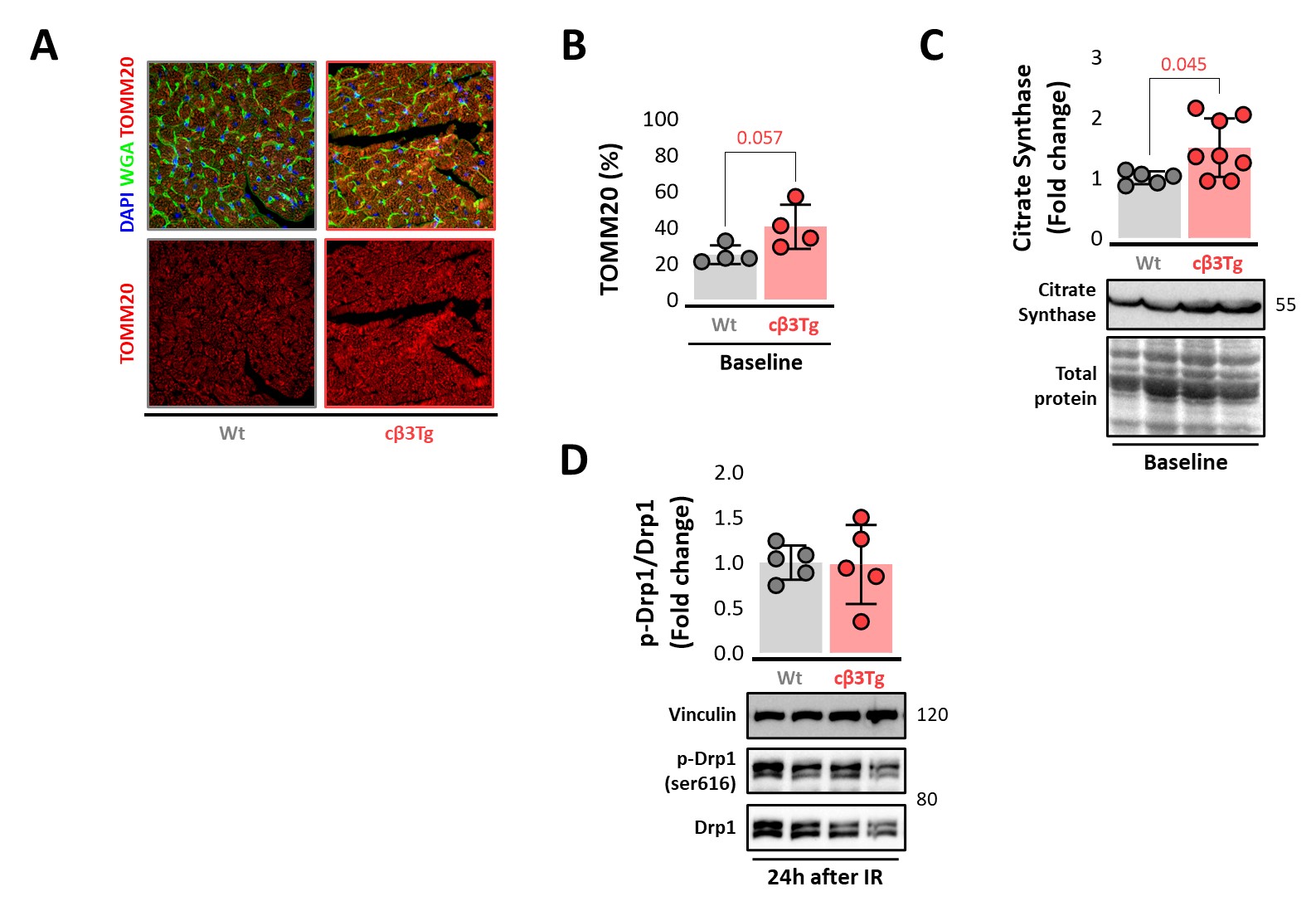

Supplement: Supplementary file 5 — Supplementary file5 (JPG 219 KB) [file 395_2024_1072_MOESM5_ESM.jpg]

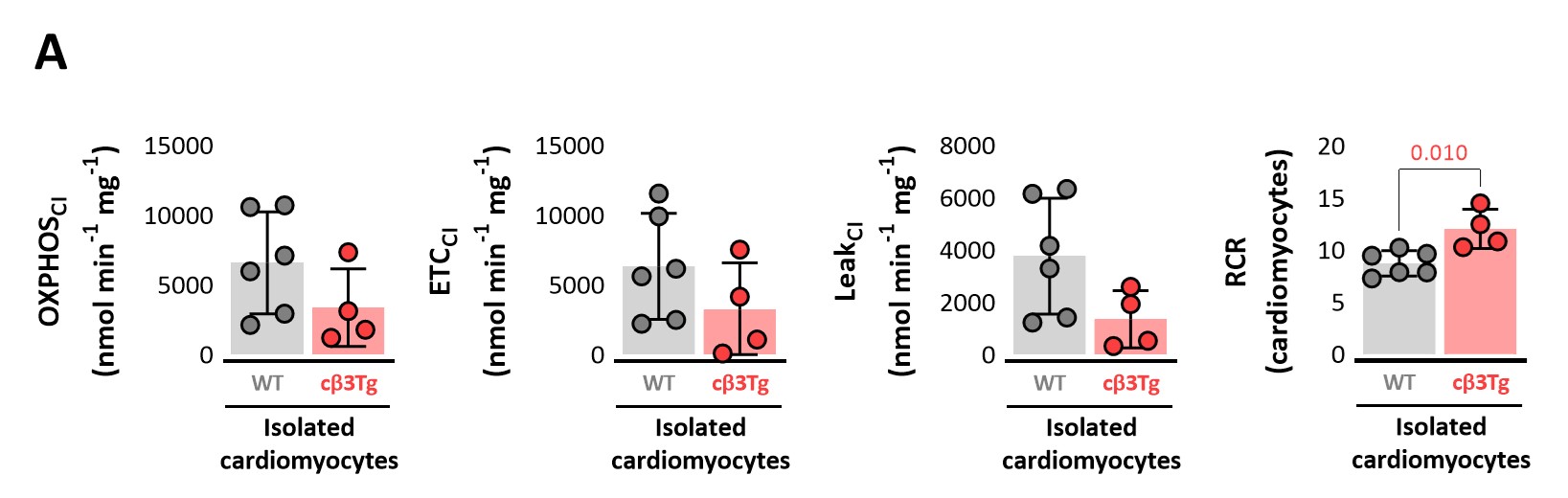

Supplement: Supplementary file 6 — Supplementary file6 (JPG 105 KB) [file 395_2024_1072_MOESM6_ESM.jpg]

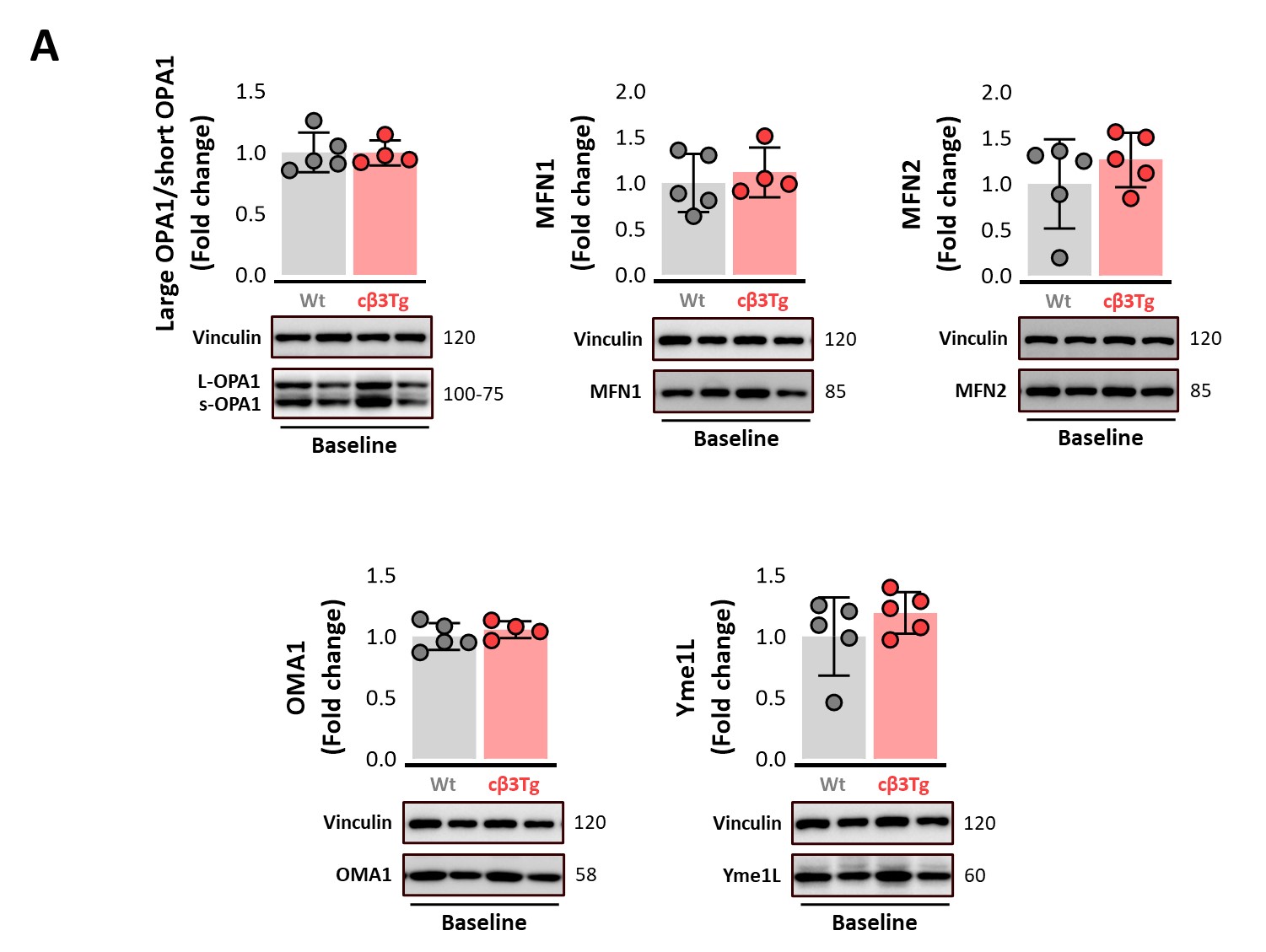

Supplement: Supplementary file 7 — Supplementary file7 (JPG 172 KB) [file 395_2024_1072_MOESM7_ESM.jpg]

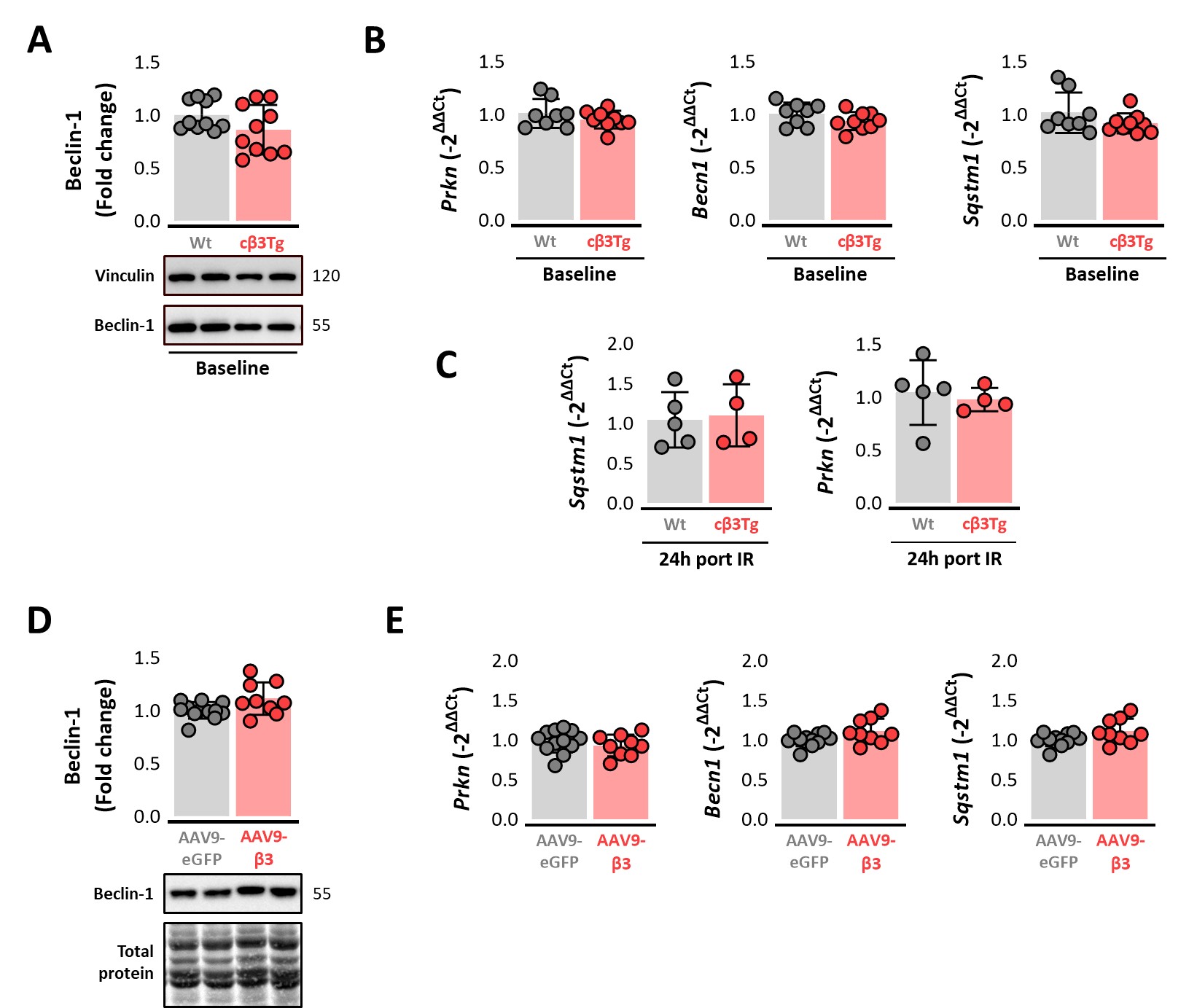

Supplement: Supplementary file 8 — Supplementary file8 (JPG 234 KB) [file 395_2024_1072_MOESM8_ESM.jpg]

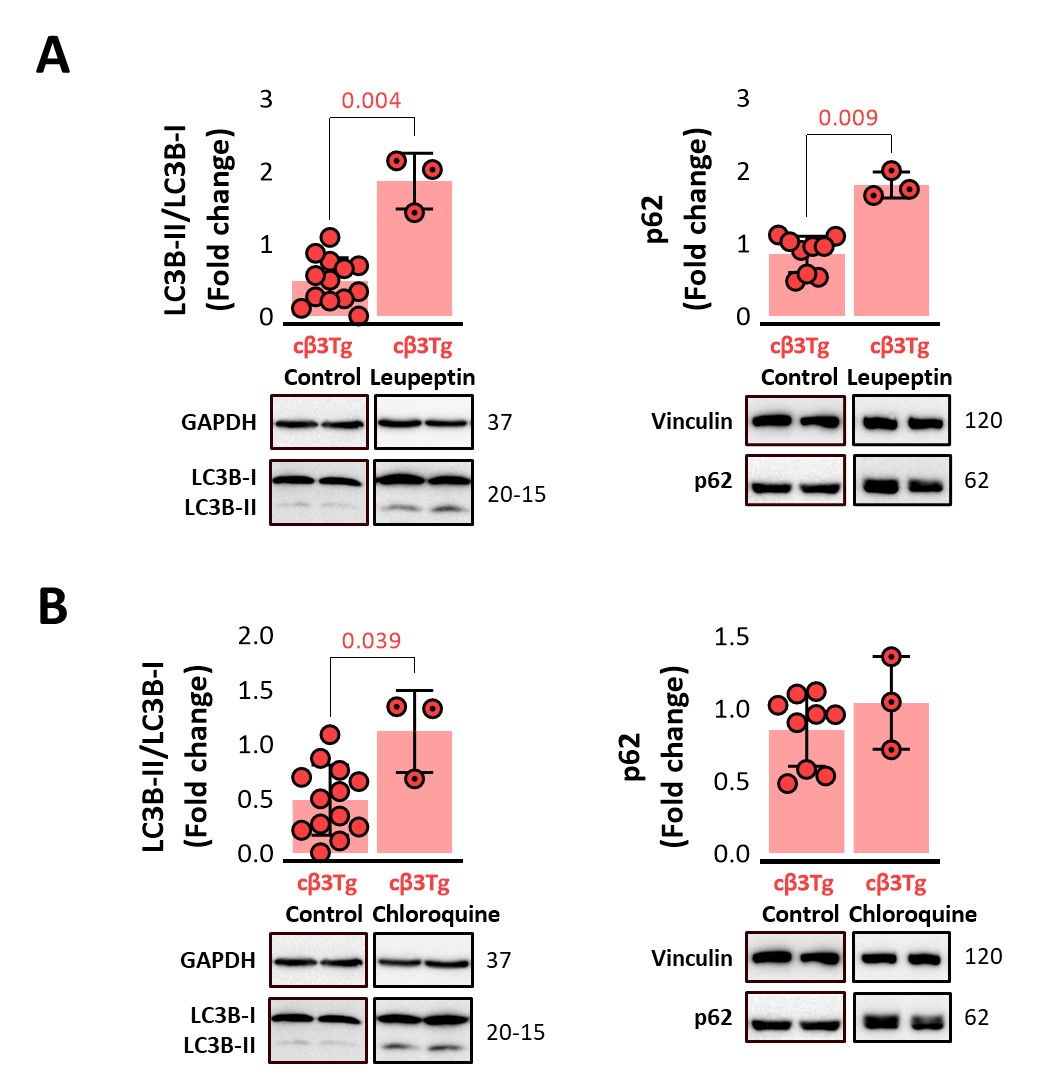

Supplement: Supplementary file 9 — Supplementary file9 (JPG 152 KB) [file 395_2024_1072_MOESM9_ESM.jpg]

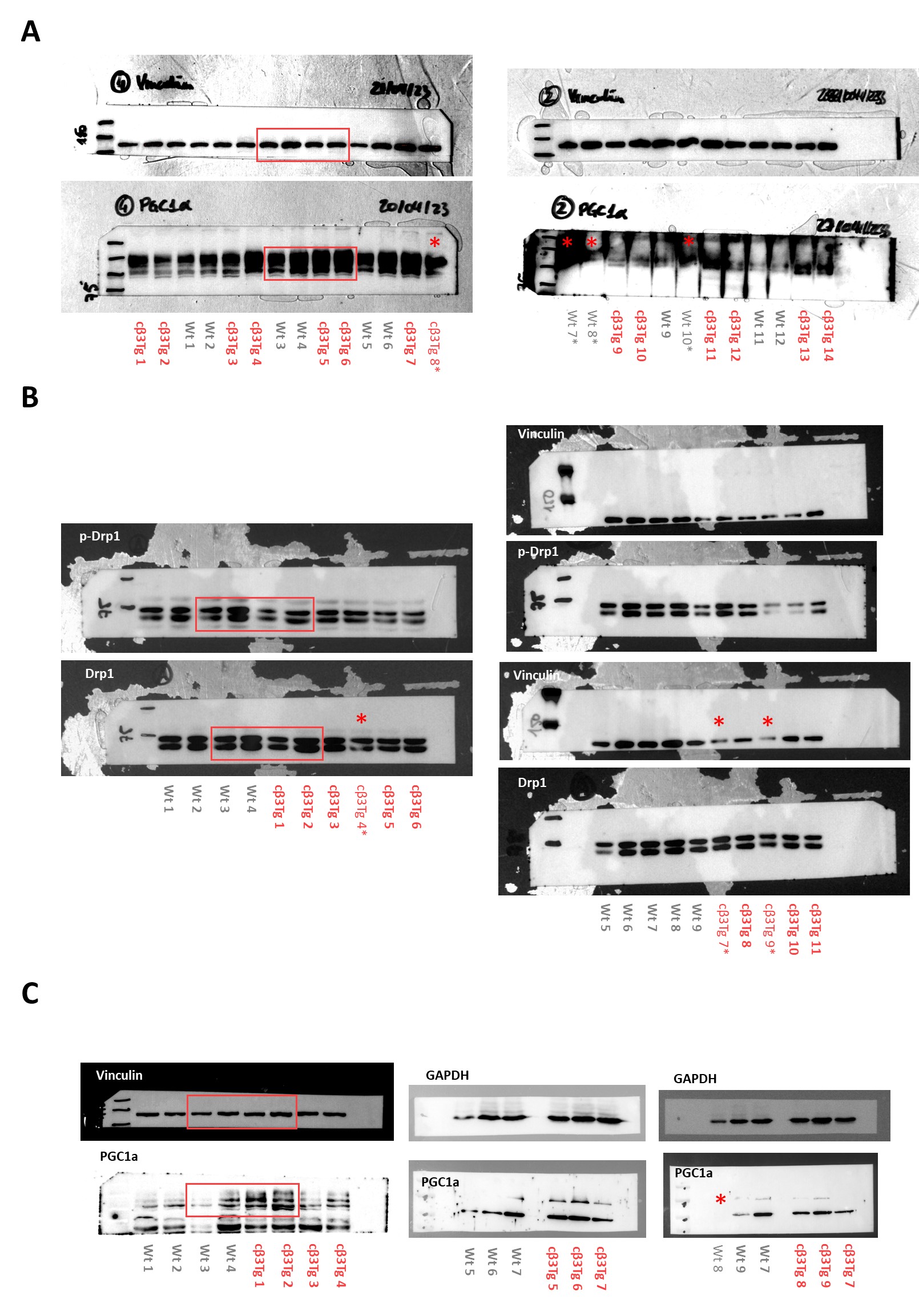

Supplement: Supplementary file 10 — Supplementary file10 (JPG 529 KB) [file 395_2024_1072_MOESM10_ESM.jpg]

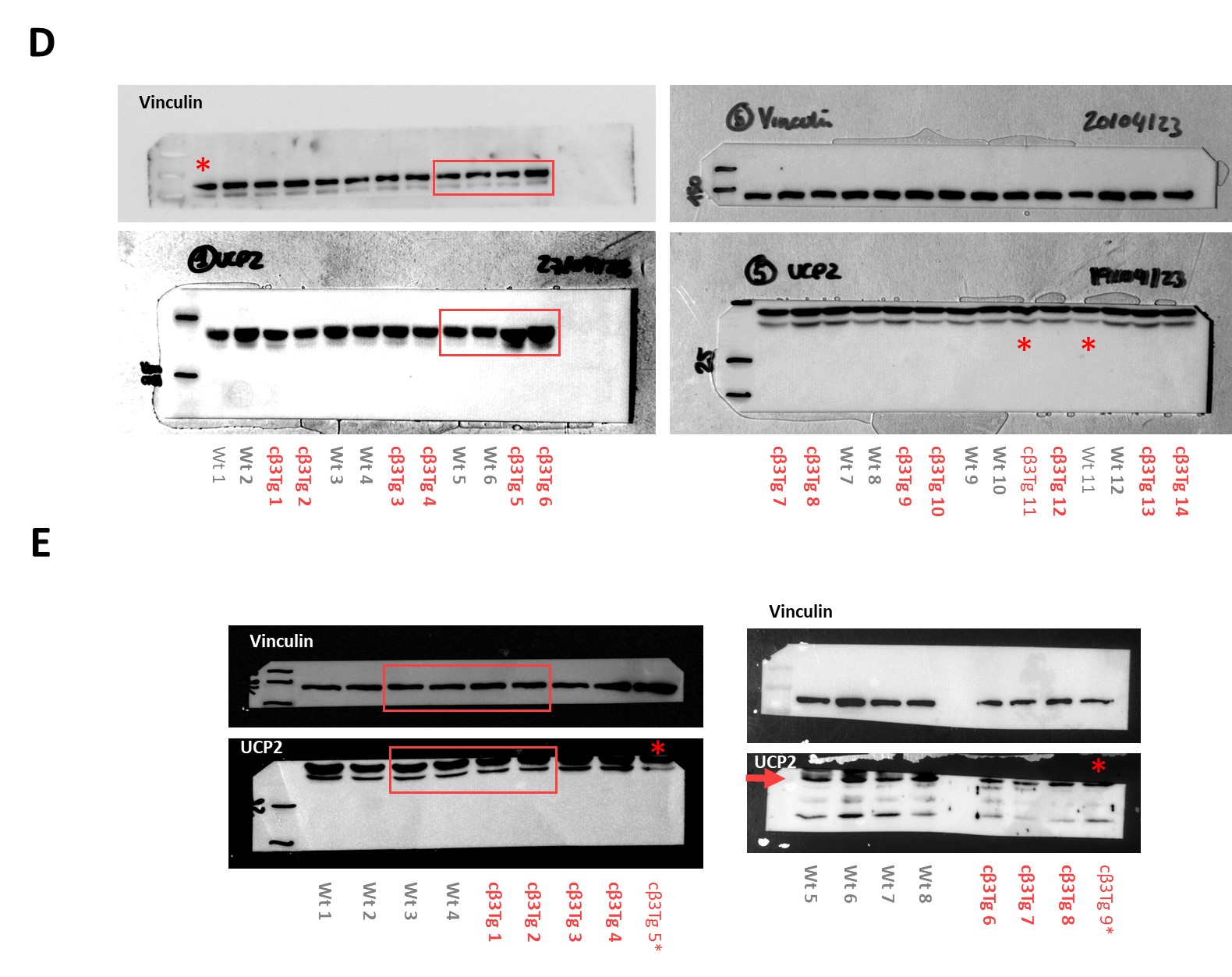

Supplement: Supplementary file 11 — Supplementary file11 (JPG 288 KB) [file 395_2024_1072_MOESM11_ESM.jpg]

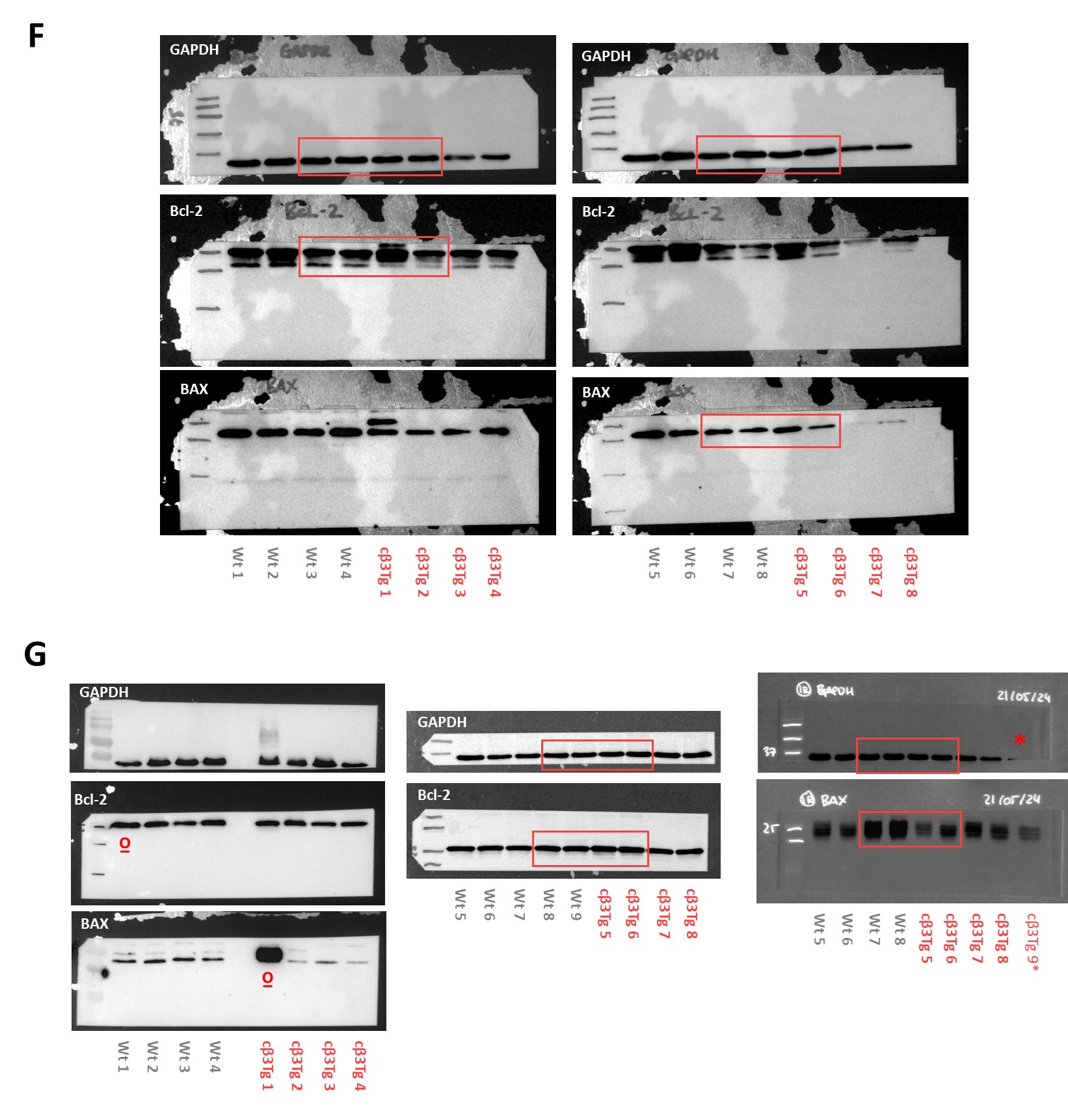

Supplement: Supplementary file 12 — Supplementary file12 (JPG 398 KB) [file 395_2024_1072_MOESM12_ESM.jpg]

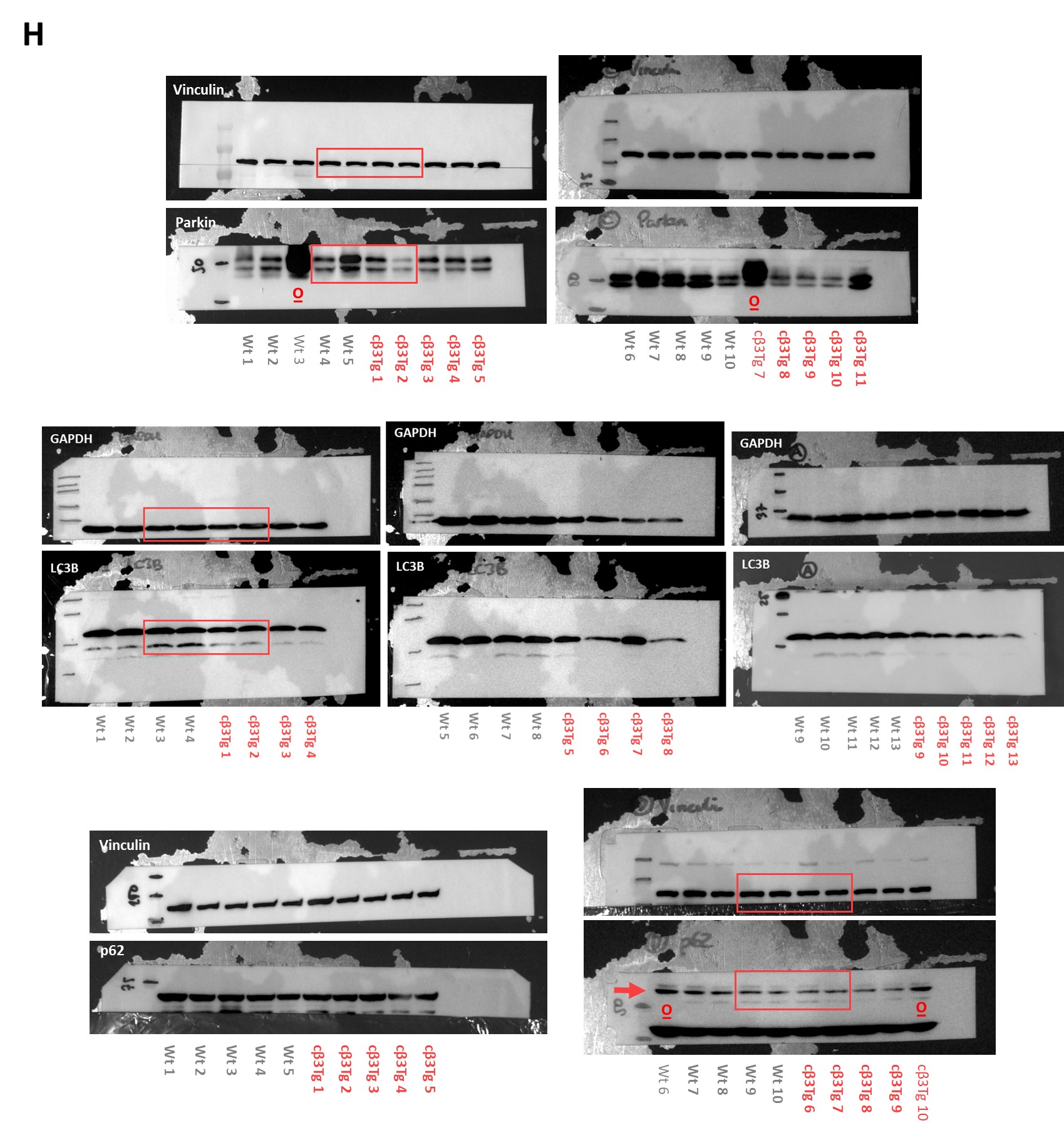

Supplement: Supplementary file 13 — Supplementary file13 (JPG 465 KB) [file 395_2024_1072_MOESM13_ESM.jpg]

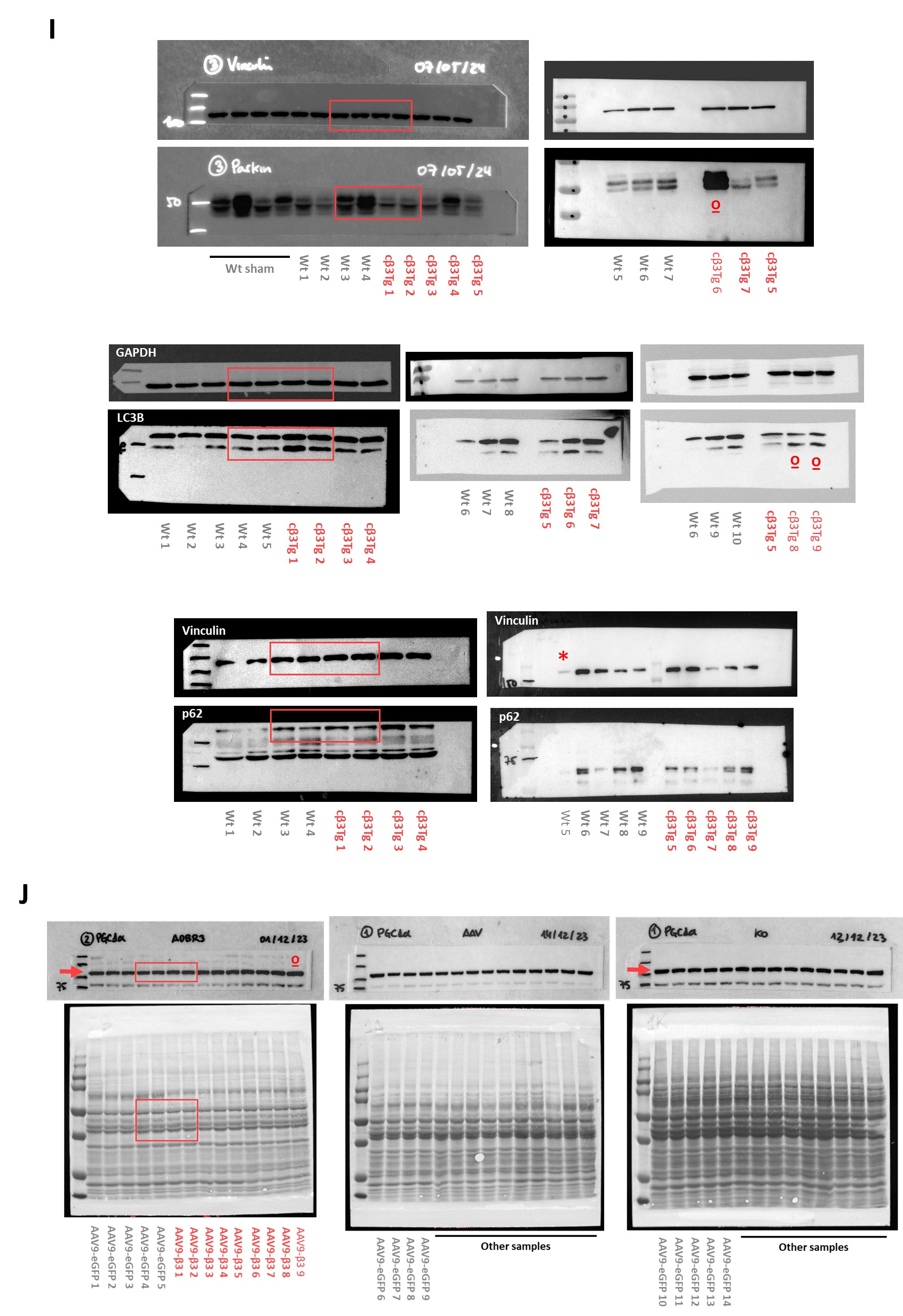

Supplement: Supplementary file 14 — Supplementary file14 (JPG 556 KB) [file 395_2024_1072_MOESM14_ESM.jpg]

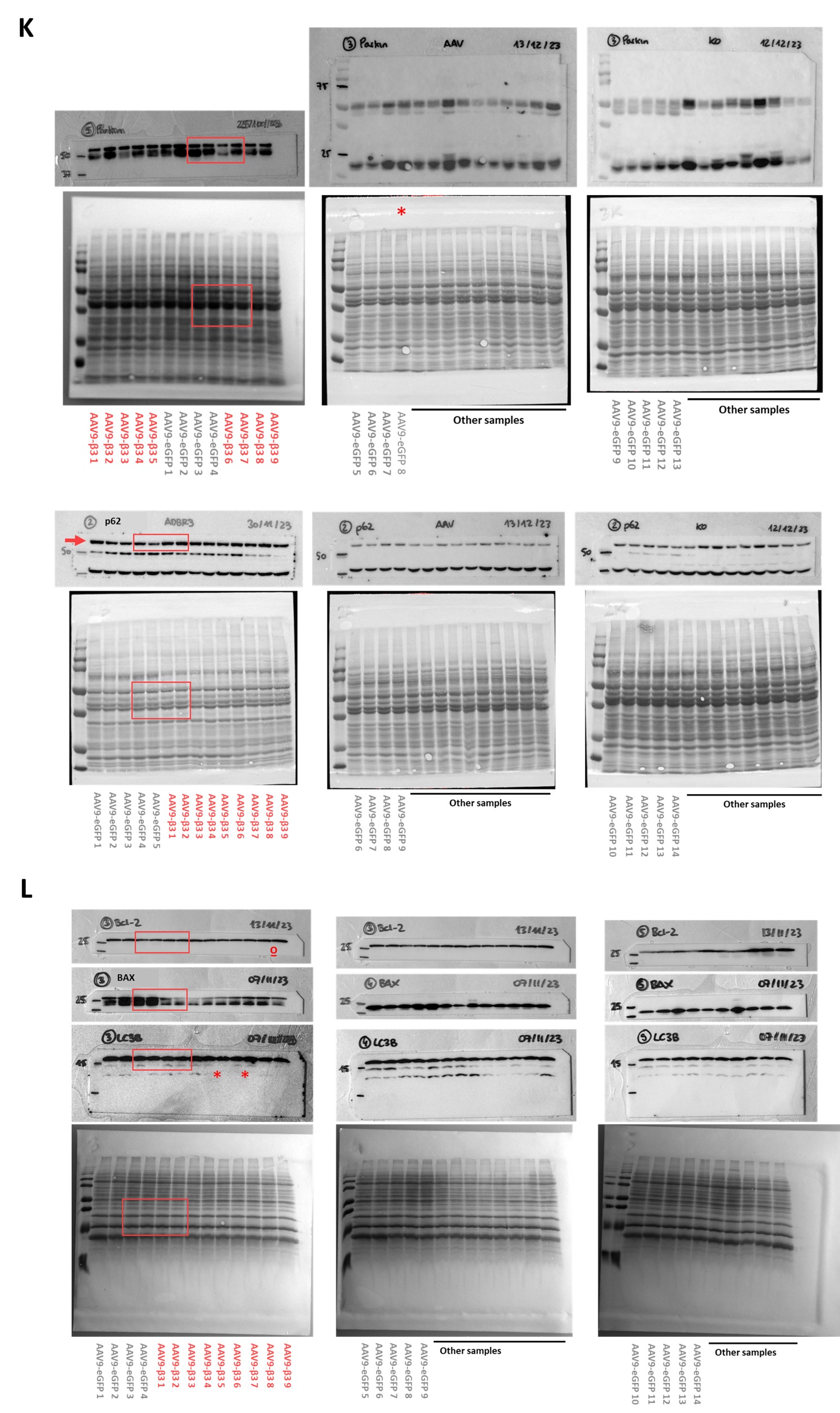

Supplement: Supplementary file 15 — Supplementary file15 (JPG 554 KB) [file 395_2024_1072_MOESM15_ESM.jpg]

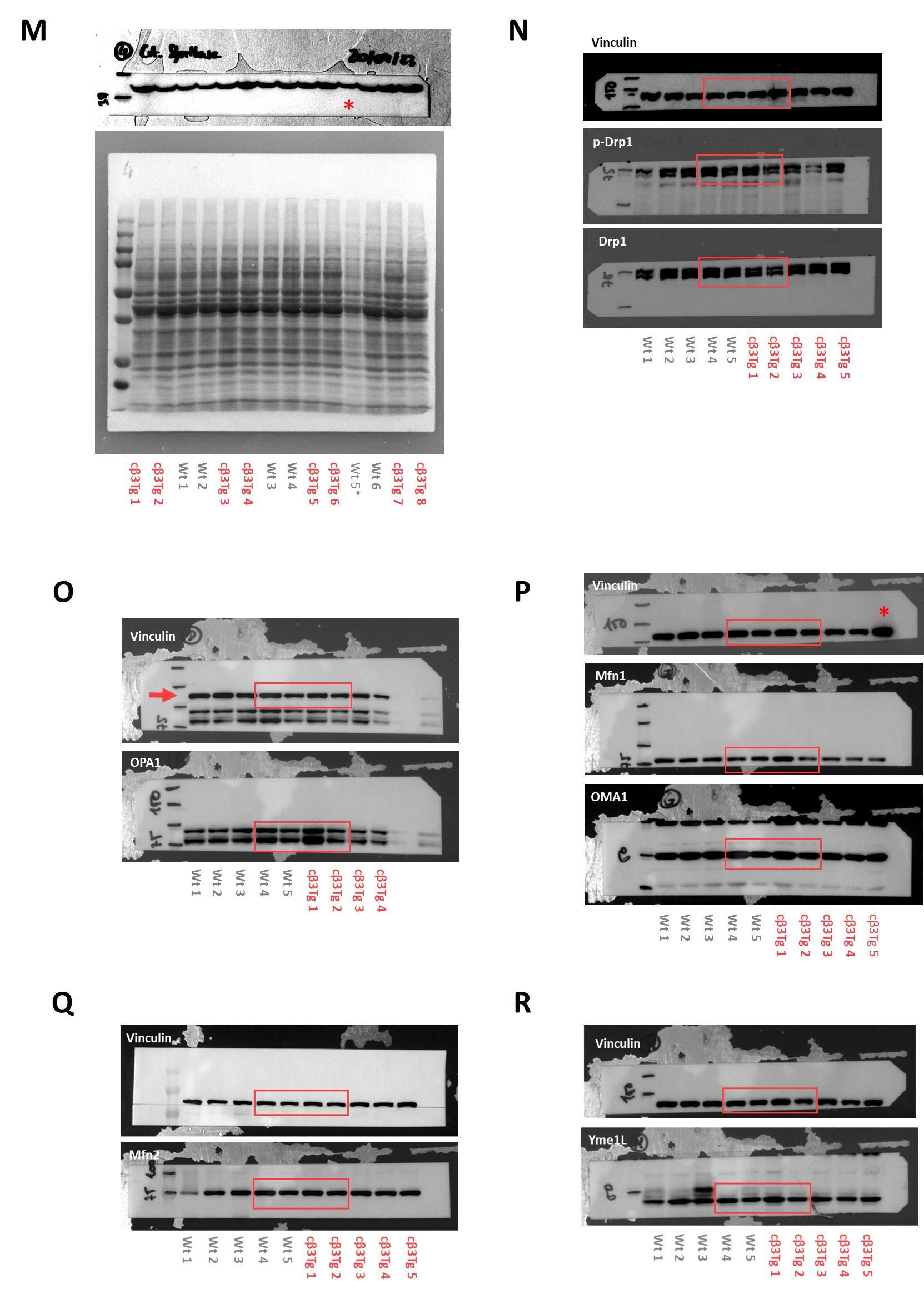

Supplement: Supplementary file 16 — Supplementary file16 (JPG 476 KB) [file 395_2024_1072_MOESM16_ESM.jpg]

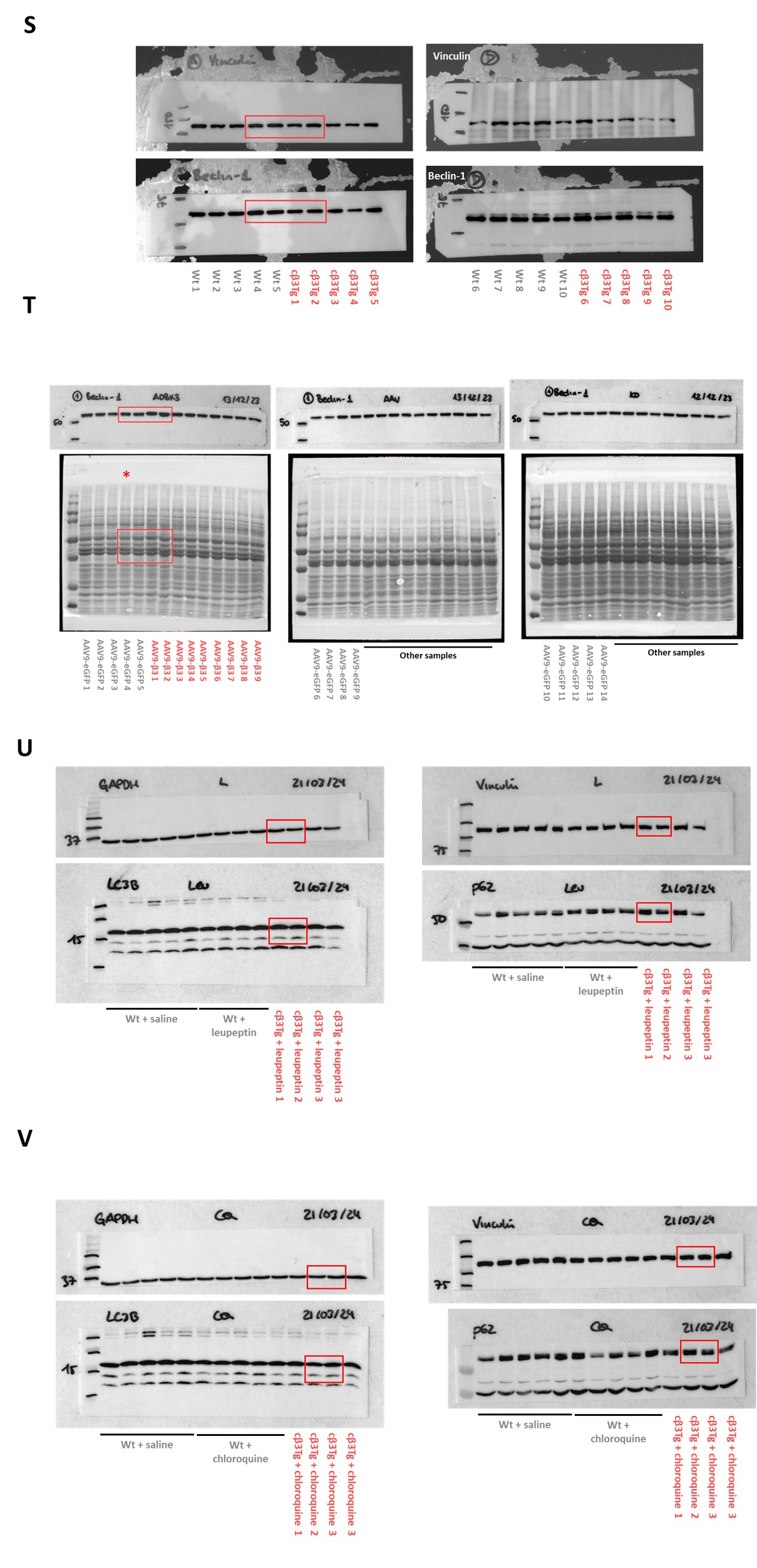

Supplement: Supplementary file 17 — Supplementary file17 (JPG 409 KB) [file 395_2024_1072_MOESM17_ESM.jpg]

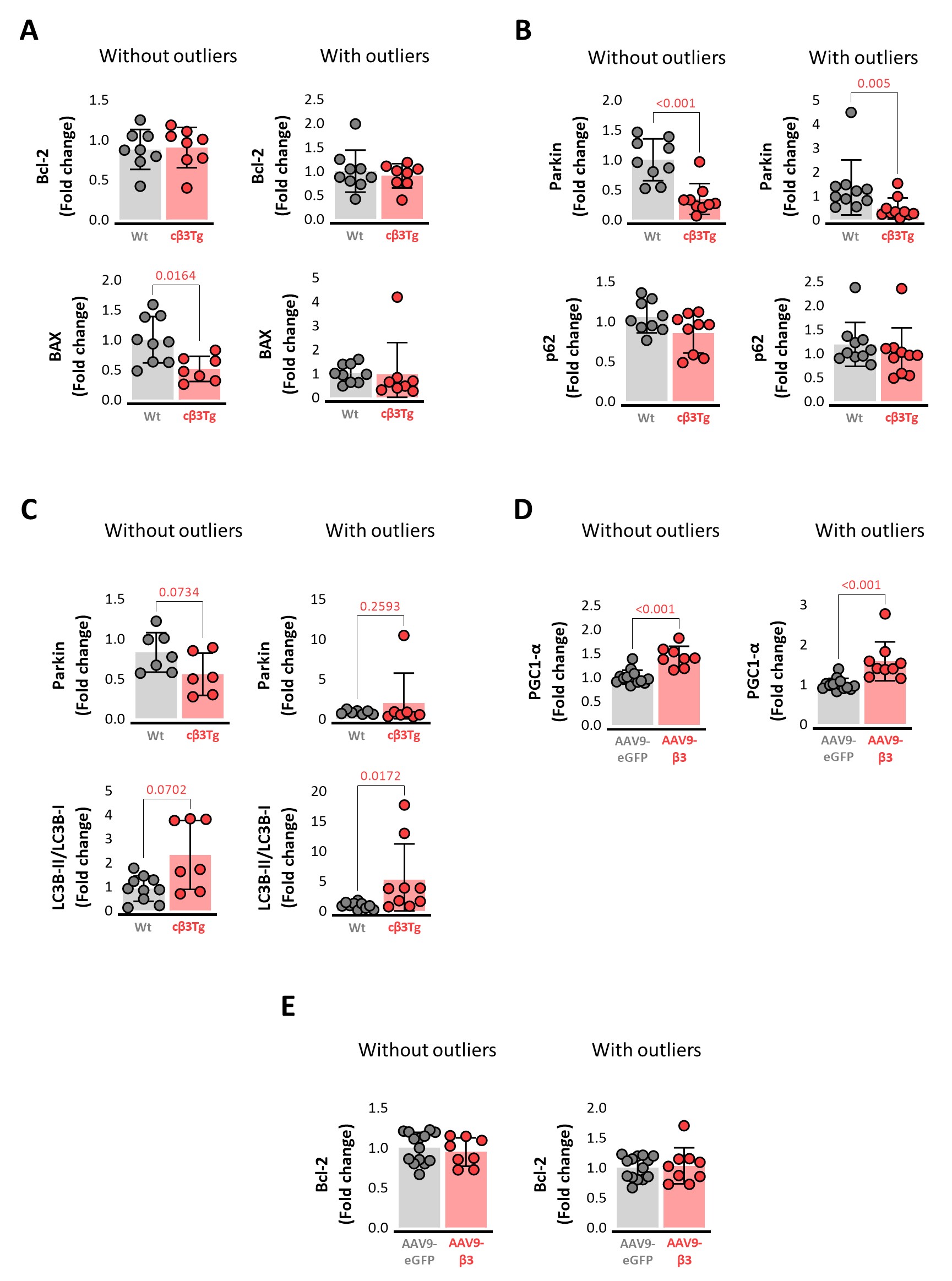

Supplement: Supplementary file 18 — Supplementary file18 (JPG 392 KB) [file 395_2024_1072_MOESM18_ESM.jpg]
