# Supplementary material for: β3-Adrenergic receptor overexpression in cardiomyocytes preconditions mitochondria to withstand ischemia–reperfusion injury
Source: Basic Res Cardiol. 2024 Aug 12;119(5):773–94. doi: 10.1007/s00395-024-01072-y (PMC11461581; doi:10.1007/s00395-024-01072-y)
Supplement: Supplementary file 19 — Supplementary file19 (DOCX 18 KB) [file 395_2024_1072_MOESM19_ESM.docx]

**Supplementary Figure 1** Generation of transgenic mice expressing human β3AR in endothelial cells and with disruption of the mouse β3AR gene in all cellular compartments. (**A-D**) Characterization of *Tie2^Cre/+^;ADRB3^tg/tg^* mice (eβ3Tg mice), expressing β3AR specifically in endothelial cells. (**A**) Representative confocal microscopy images showing vascular-restricted GFP expression in E9.5 eβ3Tg embryos but not in their wild type (*Tie2^+/+^;ADRB3^tg/tg^*) counterparts. (**B**) Representative GFP immunostaining in aortas, showing the endothelium-restricted signal in 11-13-week-old eβ3Tg mice. Scale bar, 100 µm. (**C**) hβ3AR expression in endothelial cells does not alter basal vascular tone. Arterial systolic pressure and pulse were measured using a noninvasive automated tail-cuff system in conscious mice lacking native β3AR expression (KO) and with endothelium-restricted transgenic expression of the human β3AR (e-restricted-β3). (**D**) Specific stimulation of the endothelium expressed hβ3AR in e-restricted-β3 mice induces vasodilation via NO. Thoracic aorta segments from β3KO mice (n=5) and e-restricted-β3 mice (n=7-9) were mounted on a wire myograph, and dilation was measured in response to increasing doses of the β3AR agonist mirabegron. Data are presented as the percentage of U46619-induced contraction (0.1 µM) in the absence or presence of the NOS inhibitor L-NAME (0.1 mM). *β3AR* β3-adrenergic receptor, *GFP* green fluorescent protein, *NO* nitric oxide, *NOS* nitric oxide synthase

**Supplementary Figure 2** hβ3AR expression in the mice models used. (**A-D**) RT-PCR analysis at baseline confirmed abundant expression of *ADBR3* mRNA in LV tissue compared to their respective littermate controls: β3KO mice overexpressing the human β3AR in endothelial cells (e-restricted-β3, n=6) (**A**) and β3KO mice overexpressing the human β3AR specifically in cardiomyocytes (c-restricted-β3, n=6) (**B**), compared to mice lacking native β3AR expression (KO, n=4); mice overexpressing the human β3AR in cardiomyocytes (cβ3Tg, n=9) compared to their Wt littermates (Wt, n=8) (**C**)*,* and two weeks post intravenous AAV delivery encoding human *ADBR3* gene in Wt mice (AAV9-β3*,* n=9) compared to control AAV encoding eGFP protein in Wt mice (AAV9-*eGFP,* n=9) (**D**). Data are presented as means ± SD and were analyzed by *t* test. p values are indicated on graphs when significant. *ADBR3* human β3-adrenergic receptor gene

**Supplementary Figure 3** Area at risk percentage from the left ventricle quantification in the mice models subjected to ischemia/reperfusion. Mice were subjected to left coronary artery occlusion for 45 min followed by reperfusion. Mice were sacrificed and heart samples collected 24h after reperfusion. Histological quantification of AAR is determined by Evans Blue and TTC staining on LV slices. (**A-C**) β3KO mice overexpressing the human β3AR in endothelial cells (e-restricted-β3) or in cardiomyocytes (c-restricted-β3) and their control littermates (KO), and mice overexpressing the human β3AR in cardiomyocytes (cβ3Tg) and their control littermates (Wt), were randomized to receive the β3AR-specific agonist mirabegron (1µg/kg) or vehicle by femoral-vein injection 5 min before reperfusion. e-restricted-β3 mice with mirabegron injection (n=10) and without mirabegron (n=9) versus β3KO littermate controls (n=10 and n=7) (**A**). c-restricted-β3 mice with mirabegron injection (n=8) and without mirabegron (n=7) versus β3KO littermate controls (n=8 and n=8, respectively) (**B**). cβ3Tg mice with mirabegron injection (n=9) and without mirabegron (n=8) versus littermate Wt controls (n=9 and n=8) (**C**). cβ3Tg mice and control littermates randomized to receive receive a daily oral dose of 1.15-2.02 mg chloroquine or placebo for 4 weeks before left coronary artery occlusion. cβ3Tg mice with chloroquine (n=3) and saline (n=8) versus littermate Wt controls (n=4 and n=8) (**D**). Male Wt mice were transduced with AAV9-β3 or control AAV9-*eGFP*. At 2 weeks after infection, mice underwent left coronary artery occlusion. AAV9-β3 mice (n=7) versus AAV9-*eGFP* controls (n=12). (**E**). Data are presented as means ± SD and were analyzed by *t* test or one-way or two-way ANOVA. p values are indicated on graphs when significant. *AAR* area at risk, *β3AR* β3-adrenergic receptor, *eGFP* enhanced green fluorescent protein, *LV* left ventricle, *TTC* triphenyltetrazolium chloride

**Supplementary Figure 4** Echocardiography analysis of cardiac function 7 days after IR.

**Supplementary Figure 5** Cardiac β3-adrenergic receptor overexpression increases mitochondrial number. (**A**) Western blot analysis of the mitochondrial content marker citrate synthase in LV tissue from cβ3Tg mice (n=8) and Wt littermates (n=5). (**B-C**) Confocal imaging analysis of mitochondrial area (TOMM20-positive) relative to total cell area (%) on heart sections (Wt, n=4; cβ3Tg, n=4). (**D**) LV western blot analysis of Drp-1 mediated mitochondrial fission, showing lower Drp-1 phosphorylation on ser616 in cβ3Tg hearts (Wt, n=5; cβ3Tg, n=5) 24h after IR. *DAPI* 4′,6-diamidino-2-phenylindole, *TOMM20* translocase of outer mitochondrial membrane 20, *WGA* wheat germ agglutinin, *IR* ischemia/reperfusion, *Drp1* Drp-1, dynamin-related protein 1

**Supplementary Figure 6** The mitochondrial electron transport chain is not uncoupled in β3-adrenergic receptor overexpression isolated cardiomyocytes. (**A**) Mitochondrial respiration values for OxPHOS_CI_, ETC_CI_, Leak_CI_, and RCR of isolated adult mouse cardiac myocytes from Wt mice (n=6) and from eβ3Tg mice (n=4). *CI* complex I, *OxPHOS* oxidative phosphorylation, *RCR* respiratory control ratio

**Supplementary Figure 7** Mitochondrial fusion signaling pathways were not altered in β3AR overexpression in cardiomyocytes. (**A**) Western blot data of fusion-related proteins OPA1 isoforms ratio, MTF1, MTF2, OMA1 and Yme1L of cβ3Tg mice (n=4-5) compared to their Wt control littermates (n=5) at baseline. *OPA1* mitochondrial dynamin like GTPase, *MTF1* mitofusin 1, *MTF2* mitofusin 2, *OMA1* metalloendopeptidase OMA1, *Yme1L* ATP-dependent metalloprotease

**Supplementary Figure 8** Downregulated autophagy by cardiac β3-adrenergic receptor overexpression is not explained by a defect in autophagic flux. LV from cβ3Tg and Wt control mice western blot analysis of autophagy-related protein beclin-1 at baseline (Wt, n=10; cβ3Tg, n=11) (**A**). RT-qPCR analysis of the autophagy-related genes *Sqstm1*, *Becn1*, and *Prkn* (Wt, n=8; cβ3Tg, n=8) at baseline (**B**) and 24h after IRI (Wt, n=5; cβ3Tg, n=4) (**C**). LV from AAV9-*eGFP* and AAV9-β3 infected mice western blot analysis of autophagy-related protein beclin-1 at baseline (AAV9-*eGFP*, n=12; AAV9-β3, n=9) (**D**) RT-qPCR analysis of the autophagy-related genes *Sqstm1*, *Becn1*, and *Prkn* (AAV9-*eGFP*, n=12; AAV9-β3, n=9) at baseline (**E**). *Becn1* beclin-1 gene, *Prkn*, parkin gene, *Sqstm1* p62 gene, IRI ischemia/reperfusion injury

**Supplementary Figure 9** Autophagic flux evaluation in cβ3Tg mice. Mice overexpressing the human β3AR in cardiomyocytes (cβ3Tg) and their control littermates (Wt) were subjected to a single intraperitoneal dose of leupeptin and sacrificed 45 min after injection (**A**), or chloroquine and sacrificed 4h after injection (**B**). LV western blot analysis of autophagy-related proteins LC3B and p62 show autophagic product accumulation after leupeptin (**A**) and chloroquine (**B**) dose. *LC3B* microtubule associated protein 1 light chain 3 beta, *p62* ubiquitin-binding protein p62

**Supplementary Figure 10** Uncut western blot membranes. Membranes used for data in Fig. 3D (**A**), Fig. 3F (**B**), Fig. 3H (**C**), Fig. 4D (**D**), Fig. 4I (**E**), Fig. 5B (**F**), Fig. 5E (**G**), Fig. 6B (**H**), Fig. 6D (**I**), Fig. 7E (**J**), Fig. 7G (**K-L**), Fig. 7H (**L**), Supp. Info. 5C (**M**), Supp. Info. 5D (**N**), Supp. Info. 7A (**O-R**), Supp. Info. 8A (**S**), Supp. Info. 8B (**T**), Supp. Info. 9A (**U**) and Supp. Info. 9B (**V**). Red squares indicate selected representative images. Excluded data are represented by (*) for artifacts and (º) for outliers

**Supplementary Figure 11** Western blot data comparison with and without outliers. LV western blot data comparison including or not outliers determined by Grubbs' test from Fig. 5B (**A**), Fig. 6B (**B**), Fig. 6C (**C**), Fig 7E (**D**) and Fig. 7H (**E**).
